# Supplementary material for: Examining the relationships between early childhood experiences and adolescent and young adult health status in a resource-limited population: A cohort study
Source: PLoS Med. 2021 Sep 28;18(9):e1003745. doi: 10.1371/journal.pmed.1003745 (PMC8478204; doi:10.1371/journal.pmed.1003745)
Supplement: S1 Fig — Childhood measurements (dashed lines) and approximations for adolescents (solid lines). The childhood distribution is the mean value of each child’s available measurements. Adolescents who were older than 19 years when their height was measured were assumed to be 19 years old because this is maximum age in the WHO growth reference to calculate the age- and sex-standardized HAZ. The black vertical lines indicate the mean childhood and adolescent HAZs. Of the 54.1% (630/1,025) of participants who were stunted as a child (HAZ < −2 SD from the median), 84.9% had recovered by adolescence (i.e., 535/630 had HAZ > −2), but 8.4% who had not been stunted as children became stunted as adolescents (45/535). (DOCX) [file pmed.1003745.s001.docx]

**S1 Fig**. **The distributions of HAZ from childhood measurements (dashed lines) and approximated for adolescents (solid lines).** The childhood distribution is the mean value of each child’s available measurements. Adolescents who were older than 19 years when their height was measured were assumed to be 19 years old because this is maximum age in the WHO growth reference to calculate the age and sex standardized HAZ. The black vertical lines indicate the mean childhood and adolescent HAZs. Of the 54.1% (630/1025) participants who were stunted as a child (<-2 SD from the median), 84.9% had recovered by adolescence (i.e., 535/630 had HAZ >-2), but 8.4% who had not been stunted as children became stunted as adolescents (45/535).
