# Supplementary material for: Examining the relationships between early childhood experiences and adolescent and young adult health status in a resource-limited population: A cohort study
Source: PLoS Med. 2021 Sep 28;18(9):e1003745. doi: 10.1371/journal.pmed.1003745 (PMC8478204; doi:10.1371/journal.pmed.1003745)
Supplement: S1 Table — (DOCX) [file pmed.1003745.s002.docx]

**S1** **Table Selected descriptive characteristics comparing those lost to follow-up and those re-enrolled as adolescents**

|  | **Lost to Follow-up** | **Included** | **p-value** |
| --- | --- | --- | --- |
| n | 381 | 1462 |  |
| Diarrhoea episodes/child-year (median [IQR]) | **0.54 [0.00, 1.42]** | **0.42 [0.00, 1.02]** | **0.017**^4^ |
| Pneumonia episodes/child-year (median [IQR]) | 0.00 [0.00, 0.62] | 0.00 [0.00, 0.67] | 0.433^4^ |
| Number of rooms (median [IQR]) | 2.00 [1.00, 3.00] | 2.00 [1.00, 3.00] | 0.898^4^ |
| Number of people/room (median [IQR]) | 7.00 [5.00, 10.00] | 7.00 [4.50, 10.00] | 0.193^4^ |
| Monthly household income, Rup (median [IQR])^1^ | 2999 [1499, 5999] | 2999 [1500, 5250] | 0.667^4^ |
| Sex, male (%) | **218 (57.2)** | **746 (51.0)** | **0.036**^5^ |
| Maternal education (%) |  |  | 0.133^5^ |
| Unknown | 6 (1.6) | 7 (0.5) |  |
| Illiterate | 272 (71.4) | 1031 (70.5) |  |
| ≤Matriculation | 61 (16.0) | 254 (17.4) |  |
| >Matriculation | 42 (11.0) | 170 (11.6) |  |
| Paternal education (%) |  |  | 0.285^5^ |
| Unknown | 6 (1.6) | 13 (0.9) |  |
| Illiterate | 122 (32.0) | 440 (30.1) |  |
| ≤Matriculation | 137 (36.0) | 499 (34.1) |  |
| >Matriculation | 116 (30.4) | 510 (34.9) |  |
| House type (%)^2^ |  |  | **0.002**^5^ |
| Unknown | 6 (1.6) | 6 (0.4) |  |
| Improved | 95 (24.9) | 294 (20.1) |  |
| Somewhat improved | 142 (37.3) | 667 (45.6) |  |
| Unimproved | 138 (36.2) | 495 (33.9) |  |
| Toilet type (%)^3^ |  |  | **0.032**^5^ |
| Unknown | 6 (1.6) | 9 (0.6) |  |
| Improved | 15 (3.9) | 33 (2.3) |  |
| Unimproved | 360 (94.5) | 1420 (97.1) |  |
| Drinking water treatment (%) |  |  | 0.316^5^ |
| Unknown | 6 (1.6) | 11 (0.8) |  |
| No | 169 (44.4) | 665 (45.5) |  |
| Yes | 206 (54.1) | 786 (53.8) |  |

IQR, interquartile range

^1^ Pakistani Rupees (1989)

^2^ House type based on construction materials: Improved is cement, concrete, or brick; Unimproved is mud, wood, or stone; Somewhat improved is combination.

^3^ Toilet type - Improved is flush toilet; Unimproved is open field, traditional, or pit latrine.

^4^ Kruskal-Wallis rank sum test

^5^ Chi-squared test
