# Supplementary material for: Examining the relationships between early childhood experiences and adolescent and young adult health status in a resource-limited population: A cohort study
Source: PLoS Med. 2021 Sep 28;18(9):e1003745. doi: 10.1371/journal.pmed.1003745 (PMC8478204; doi:10.1371/journal.pmed.1003745)
Supplement: S2 Table — (DOCX) [file pmed.1003745.s003.docx]

**S2 Table:** **Univariate relationships predicting the squared Raven’s T-scores assuming a linear regression between each variable and the squared Raven’s T**

| **Variable** | **Category** | **Estimate** | **95% CI** | **p-value** |
| --- | --- | --- | --- | --- |
| *Childhood variables* |  |  |  |  |
| Sex | Female |  |  | **<0.001** |
|  | Male | **280** | **(180, 370)** |  |
| Mean HAZ | z-score | 37 | (-6.6, 80) | 0.096 |
| Diarrhoea | Episodes/Follow-Up | -800 | (-2200, 640) | 0.276 |
| Maternal Education | Illiterate |  |  | **<0.001** |
|  | <=Matriculation | **370** | **(260, 480)** |  |
|  | >Matriculation | **870** | **(580, 1200)** |  |
| Paternal Education | Illiterate |  |  | **<0.001** |
|  | <=Matriculation | **270** | **(160, 390)** |  |
|  | >Matriculation | **610** | **(470, 750)** |  |
| Maternal Occupation | Housewife |  |  | **<0.001** |
|  | Other | **710** | **(520, 900)** |  |
| Paternal Occupation | High |  |  | **<0.001** |
|  | Medium | -130 | (-270, 2.1) |  |
|  | Low | **-420** | **(-550, -280)** |  |
|  | Not earning | **-280** | **(-470, -89)** |  |
| Parental Income^1^ | Rup. 0 |  |  | **<0.001** |
|  | Rup. 1-499 | -180 | (-420, 59) |  |
|  | Rup. 500-999 | -80 | (-260, 100) |  |
|  | Rup. 1000-1999 | 24 | (-140, 180) |  |
|  | Rup. 2000-3999 | **320** | **(150, 500)** |  |
|  | Rup. 4000+ | **310** | **(57, 570)** |  |
| House Type^2^ | Improved |  |  | **<0.001** |
|  | Unimproved | **-400** | **(-530, -260)** |  |
|  | Somewhat improved | **-200** | **(-330, -70)** |  |
|  | Other | 95 | (-970, 1200) |  |
| Number of Rooms | Count | **420** | **(310, 540)** | **<0.001** |
| Toilet Type^3^ | Flush Toilet |  |  | **0.003** |
|  | Open Field | **-630** | **(-1100, -150)** |  |
|  | Traditional Latrine | **-520** | **(-830, -220)** |  |
|  | Pit Latrine | **-650** | **(-980, -320)** |  |
|  | Other | -210 | (-1300, 900) |  |
| Household Density | People/Rooms | **-160** | **(-220, -91)** | **<0.001** |
|  |  |  |  |  |
| *Adolescent follow-up* |  |  |  |  |
| Age | Years | -9.4 | (-24, 4.8) | 0.192 |
| Age at Moving | Did Not Move |  |  | **<0.001** |
|  | Move Age 0–15 | **408** | **(249, 567)** |  |
|  | Move Age >15 | **274** | **(156, 391)** |  |
| Waist Girth | cm | **5.2** | **(0.11, 10)** | **0.045** |
| Height | cm | **21** | **(16, 26)** | **<0.001** |
| Weight | kg | **13** | **(7.6, 18)** | **<0.001** |
| BMI |  | -4.2 | (-20, 11) | 0.593 |
| Systolic BP | mm/Hg | 3.4 | (-1.4, 8.3) | 0.163 |
| Diastolic BP | mm/Hg | -0.46 | (-6.2, 5.3) | 0.878 |
| Hypertension | Normal |  |  | 0.427 |
|  | Elevated | 170 | (-120, 460) |  |
|  | Hypertension I | 93 | (-65, 250) |  |
|  | Hypertension II | -83 | (-430, 260) |  |
| SRCH | Excellent |  |  | **<0.001** |
|  | Good | -160 | (-350, 33) |  |
|  | Satisfactory | **-330** | **(-510, -160)** |  |
|  | Poor | **-570** | **(-770, -370)** |  |
|  | Very Poor | -410 | (-2200, 1400) |  |
| SRPH | Excellent |  |  | **<0.001** |
|  | Good | **-390** | **(-580, -200)** |  |
|  | Satisfactory | **-540** | **(-730, -350)** |  |
|  | Poor | **-660** | **(-850, -460)** |  |
|  | Very Poor | -500 | (-1200, 160) |  |
| Lifetime Health Problems | None |  |  | 0.560 |
|  | Some | 33 | (-79, 150) |  |
| Current Location | Oshikhandass |  |  | **<0.001** |
|  | Islamabad | **730** | **(560, 900)** |  |
|  | Lahore | **590** | **(270, 900)** |  |
|  | Karachi | **310** | **(150, 460)** |  |
|  | Quetta | 550 | (-130, 1200) |  |
|  | Peshawar | 100 | (-700, 900) |  |
|  | Faisalabad | -380 | (-1600, 890) |  |
|  | Other | -73 | (-230, 84) |  |
| Primary Language | Burushaski |  |  | **<0.001** |
|  | Other | -67 | (-640, 510) |  |
|  | Shina | **-440** | **(-550, -340)** |  |
| English Spoken | No |  |  | **<0.001** |
|  | Yes | **850** | **(750, 950)** |  |
| Education Level | Years | **130** | **(110, 140)** | **<0.001** |
| Repeat Class | Yes |  |  | **0.002** |
|  | No | **150** | **(55, 250)** |  |
| Employment | Unemployed |  |  | **<0.001** |
|  | Employed | **380** | **(220, 540)** |  |
|  | Student | **550** | **(410, 690)** |  |
| Married | Yes |  |  | **<0.001** |
|  | No | **350** | **(200, 490)** |  |

Bold values indicate terms that did not include zero in the 95% confidence interval and were significant at the p<0.05 level.

HAZ, length/height-for-age *z* score; BMI, body mass index; BP, blood pressure; SRCH, self-reported current health status, SRPH, self-reported past health (between ages 5-15)

^1^ Pakistani Rupees (1989)

^2^ House type based on construction materials: Improved is cement, concrete, or brick; Unimproved is mud, wood, or stone; Somewhat improved is combination.

^3^ Toilet type - Improved is flush toilet; Unimproved is open field, traditional, or pit latrine.
