# Supplementary material for: Examining the relationships between early childhood experiences and adolescent and young adult health status in a resource-limited population: A cohort study
Source: PLoS Med. 2021 Sep 28;18(9):e1003745. doi: 10.1371/journal.pmed.1003745 (PMC8478204; doi:10.1371/journal.pmed.1003745)
Supplement: S3 Table — (DOCX) [file pmed.1003745.s004.docx]

**S3 Table.** **Characteristics of the cohort according to whether adolescents spoke English**

|  | **English Spoken** | |  |
| --- | --- | --- | --- |
| **Variable** | **No** | **Yes** | **p-value** |
| *n* | 1072 | 390 |  |
| *mean* |  |  |  |
| Years of Education | 10.3 | 13.1 | 0.08^2^ |
| Household Density | 2.7 | 2.6 | 0.01^3^ |
| ***%*** |  |  |  |
| Moved away from Oshikhandass | 25.5 | 62.6 | <0.01^4^ |
| Mother was Literate/Received Formal Education | 24.8 | 41.1 | 0.02^4^ |
| Father was Literate/Received Formal Education | 65.6 | 80.8 | 0.02^4^ |
| Childhood Household Income ≥2000Rup.^1^ | 21.6 | 37.0 | 0.03^4^ |
| Improved House | 17.6 | 26.5 | 0.18^4^ |
| Improved Toilet | 15.9 | 19.3 | 0.66^4^ |

^1^ Pakistani Rupees (1989)

^2^ *t-*test

^3^ Kruskal-Wallis rank sum test

^4^ Chi-squared test
