# Supplementary material for: Examining the relationships between early childhood experiences and adolescent and young adult health status in a resource-limited population: A cohort study
Source: PLoS Med. 2021 Sep 28;18(9):e1003745. doi: 10.1371/journal.pmed.1003745 (PMC8478204; doi:10.1371/journal.pmed.1003745)
Supplement: S4 Table — Showing coefficients from linear models for Raven’s T score, BMI, and blood pressure; a Poisson model for education (log count); and a multinomial for employment status (log odds), all as a function of childhood diarrhea episodes. Variable inclusion was based on the Bayesian network. (DOCX) [file pmed.1003745.s005.docx]

**S4 Table.** **Table Regression models of the primary outcomes.** Showing coefficients from linear models for Raven’s scores, BMI, and blood pressure; a Poisson model for education [log count]; and a multinomial for employment status [log odds] all as a function of childhood diarrhea episodes. Variable inclusion was based on the Bayesian Network.

| **Term** | **Raven's scores (squared T score)** | **BMI (kg/cm2)** | **Blood Pressure (mm Hg)** | | **Education (years)** | **Employment (relative to Unemployed, log odds)** | |
| --- | --- | --- | --- | --- | --- | --- | --- |
|  |  |  | **Systolic** | **Diastolic** |  | **Employed** | **Student** |
|  | ***Diarrhoea episodes/child-year (n = 1165)*** | | | | | | |
| Height (cm) | 0.14 [ 0.08; 0.19]^*^ | -0.27 [-0.27; -0.26]^*^ |  |  |  |  |  |
| Age (years) | -0.42 [ -0.58; -0.26]^*^ |  |  |  | 0.03 [ 0.02; 0.03]^*^ |  |  |
| Education (years) | 0.97 [ 0.73; 1.21]^*^ |  |  |  |  |  |  |
| English Spoken (Yes) | 5.97 [ 4.64; 7.30]^*^ | 0.06 [ 0.01; 0.12]^*^ |  |  | 0.16 [ 0.12; 0.20]^*^ |  |  |
| Diarrhoea sqrt (episodes/child-year) | -3.05 [-21.78; 15.69] | 0.47 [-0.35; 1.30] | -10.92 [-30.35; 8.50] | -6.45 [-17.85; 4.95] | -0.35 [-0.96; 0.27] | -8.20 [-15.42; -0.97]^*^ | -4.46 [-10.35; 1.43] |
| Weight (kg) |  | 0.37 [ 0.37; 0.38]^*^ | 0.40 [ 0.34; 0.45]^*^ |  |  |  |  |
| Sex (Male) |  | 0.01 [-0.06; 0.07] |  |  |  | 2.52 [ 2.06; 2.99]^*^ | 1.44 [ 1.04; 1.84]^*^ |
| BP (systolic, mm Hg) |  |  |  | 0.65 [ 0.62; 0.68]^*^ |  |  |  |
| Employment  (reference: unemployed) |  |  |  |  |  |  |  |
| Employed |  |  |  |  | 0.06 [ 0.00; 0.12]^*^ |  |  |
| Student |  |  |  |  | 0.20 [ 0.14; 0.26]^*^ |  |  |

* Indicates terms that did not include 0 in the 95% confidence interval

BMI, body mass index; BP, blood pressure
