# Supplementary material for: Examining the relationships between early childhood experiences and adolescent and young adult health status in a resource-limited population: A cohort study
Source: PLoS Med. 2021 Sep 28;18(9):e1003745. doi: 10.1371/journal.pmed.1003745 (PMC8478204; doi:10.1371/journal.pmed.1003745)
Supplement: S5 Table — Showing coefficients from linear models for Raven’s T score, BMI, and blood pressure; a Poisson model for education (log count); and a multinomial for employment status (log odds), all as a function of childhood pneumonia episodes. Variable inclusion was based on the Bayesian network. (DOCX) [file pmed.1003745.s006.docx]

**S5 Table Regression models of the primary outcomes**. Showing coefficients from linear models for Raven’s scores, BMI and blood pressure; a Poisson model for education [log count]; and a multinomial for employment status [log odds] all as a function of childhood pneumonia episodes. Variable inclusion was based on the Bayesian Network.

| **Term** | **Raven's scores (squared T score)** | **BMI (kg/cm2)** | **Blood Pressure (mm Hg)** | | **Education (years)** | **Employment (relative to Unemployed, log odds)** | |
| --- | --- | --- | --- | --- | --- | --- | --- |
|  |  |  | **Systolic** | **Diastolic** |  | **Employed** | **Student** |
|  |  |  |  |  |  |  |  |
|  | ***Pneumonia episodes/child-year (n = 857)*** | | | | | | |
| Height (cm) | 0.15 [ 0.08; 0.21]^*^ | -0.26 [-0.26; -0.25]^*^ |  |  |  |  |  |
| Age (years) | -0.65 [ -0.91; -0.38]^*^ |  |  |  | 0.04 [ 0.03; 0.05]^*^ |  |  |
| Education (years) | 1.18 [ 0.87; 1.49]^*^ |  |  |  |  |  |  |
| English Spoken (Yes) | 6.17 [ 4.63; 7.71]^*^ | 0.07 [ 0.01; 0.12]^*^ |  |  | 0.12 [ 0.07; 0.17]^*^ |  |  |
| Pneumonia sqrt (episodes/child-year) | -0.11 [-21.87; 21.64] | 0.16 [-0.68; 1.00] | -0.50 [-22.45; 21.45] | -5.98 [-18.89; 6.93] | -0.27 [-1.02; 0.49] | -4.79 [-16.23; 6.65] | 6.08 [ -2.61; 14.77] |
| Weight (kg) |  | 0.37 [ 0.37; 0.38]^*^ | 0.38 [ 0.32; 0.45]^*^ |  |  |  |  |
| Sex (Male) |  | -0.03 [-0.10; 0.03] |  |  |  | 3.18 [ 2.47; 3.90]^*^ | 1.61 [ 1.05; 2.17]^*^ |
| BP (systolic, mm Hg) |  |  |  | 0.65 [ 0.61; 0.69]^*^ |  |  |  |
| Employment  (reference: unemployed) |  |  |  |  |  |  |  |
| Employed |  |  |  |  | 0.02 [-0.06; 0.11] |  |  |
| Student |  |  |  |  | 0.20 [ 0.12; 0.27]^*^ |  |  |
|  |  |  |  |  |  |  |  |

* Indicates terms that did not include 0 in the 95% confidence interval

BMI, body mass index; BP, blood pressure
