# Supplementary material for: Examining the relationships between early childhood experiences and adolescent and young adult health status in a resource-limited population: A cohort study
Source: PLoS Med. 2021 Sep 28;18(9):e1003745. doi: 10.1371/journal.pmed.1003745 (PMC8478204; doi:10.1371/journal.pmed.1003745)
Supplement: S6 Table — Forty-five studies identified using 2 search strategies of the PubMed (US National Library of Medicine) database were manually filtered by the first and last author for relevance to this study. Search 1: (cohort OR longitudinal) AND (childhood OR adolescence) AND (cognitive OR cognition) AND (diarrhea OR diarrhoea OR pneumonia OR “childhood illness*”) AND (BMI OR “health progression” OR growth OR “child development” OR “childhood development”). Search 1 yielded 21 studies. Search 2: ((child* OR infant OR adolescen*) AND (cohort* OR longitudinal OR followup OR “follow up”)) AND cogniti* AND ((“human capital” OR “human capacit*” OR potential OR “child development” OR “health progression” OR “adolescent development”) AND ((adult* OR adoles*) AND outcome*))) AND (undernutrition OR stunt* OR diarrhea OR diarrhoea OR pneumonia OR “childhood illness*”). Search 2 yielded 34 studies. And 30 more studies were identified based on reviewers’ comments. (DOCX) [file pmed.1003745.s007.docx]

| **S6 Table: Literature Search for Research in Context** | | |  |  |  |  |  |  |  |
| --- | --- | --- | --- | --- | --- | --- | --- | --- | --- |
|  |  |  |  |  |  |  |  |  |  |
| Forty-five studies identified using the following two search strategies were manually filtered by the first and last author for relevance to this study: | | | | | | |  |  |  |
| (cohort OR longitudinal) AND (childhood OR adolescence) AND (cognitive OR cognition) AND (diarrhea OR diarrhoea OR pneumonia | | | | | | |  |  |  |
| OR 'childhood illness*")AND (BMI OR “health progression” OR growth OR “child development” OR “childhood development”) = 21 | | | | | | |  |  |  |
| ((child* OR infant OR adolescen*) AND (cohort* OR longitudinal OR followup OR “follow up”)) AND cogniti* AND ((“human capital” OR “human | | | | | | |  |  |  |
| capacit*" OR potential OR “child development” OR “health progression” OR “adolescent development”) AND ((adult* OR adoles*) AND outcome*))) | | | | | | |  |  |  |
| AND (undernutrition OR stunt* OR diarrhea OR diarrhoea OR pneumonia OR “childhood illness*”) = 34 | | | | | |  |  |  |  |
| And 30 based on reviewers' comments | | |  |  |  |  |  |  |  |
|  |  |  |  |  |  |  |  |  |  |
| **Year** | **First Author** | **Title** | **Age** | **Topic** | **Publication Title** | **DOI** | **Pages** | **Issue** | **Volume** |
| 2017 | Adler, S | Symptoms and risk factors of Cryptosporidium hominis infection in children: data from a large waterborne outbreak in Sweden. | Childhood | Infection | Parasitology research | 10.1007/s00436-017-5558-z | 2613-2618 | 10 | 116 |
| 2011 | Ajjampur, SSR | Effect of cryptosporidial and giardial diarrhoea on social maturity, intelligence and physical growth in children in a semi-urban slum in south India. | Childhood | Infection | Annals of tropical paediatrics | 10.1179/1465328111Y.0000000003 | 205-212 | 3 | 31 |
| 2020 | Alam, MA | Impact of early-onset persistent stunting on cognitive development at 5 years of age: Results from a multi-country cohort study | Childhood | Cognition | PLoS One | doi: 10.1371/journal.pone.0227839 | 340-57 | 371 | 9609 |
| 2017 | Alderman, H | Evidence of Impact of Interventions on Growth and Development during Early and Middle Childhood. | Childhood | Growth | Child and Adolescent Health and Development | 10.1596/978-1-4648-0423-6 |  |  |  |
| 2014 | Alderman, H | Supplemental feeding during pregnancy compared with maternal supplementation during lactation does not affect schooling and cognitive development through late adolescence. | Adolescence | Nutrition | The American j of clinical nutrition | 10.3945/ajcn.113.063404 | 122-129 | 1 | 99 |
| 2019 | Aoyagi, S | Does maternal postpartum depression affect children's developmental outcomes? | Childhood | Depression | The jof obstetrics and gynaecology research | 10.1111/jog.14064 | 1809-1820 | 9 | 45 |
| 2002 | Berkman, DS | Effects of stunting, diarrhoeal disease, and parasitic infection during infancy on cognition in late childhood: a follow-up study. | Childhood | Growth/ Morbidity | Lancet | 10.1016/S0140-6736(02)07744-9 | 564-571 | 9306 | 359 |
| 2018 | Berry, D | Otitis media and respiratory sinus arrhythmia across infancy and early childhood: Polyvagal processes? | Childhood | Morbidity | Developmental psychology | 10.1037/dev0000488 | 1709-1722 | 9 | 54 |
| 2004 | Bhargava, SK | Relation of serial changes in childhood body-mass index to impaired glucose tolerance in young adulthood | Adult | Morbidity | N Eng J Med | doi: 10.1056/NEJMoa035698 | 865-75 | 350 | 9 |
| 2017 | Black, MM | Early childhood development coming of age: science through the life course | Childhood | Socio-Demographic | Lancet | doi: 10.1016/s0140-6736(16)31389-7 | 77-90 | 389 | 10064 |
| 2016 | Casale, D | Recovery from stunting and cognitive outcomes in young children: evidence from the South African Birth to Twenty Cohort Study. | Childhood | Nutrition | J of developmental origins of health and disease | 10.1017/S2040174415007175 | 163-171 | 2 | 7 |
| 2014 | Connelly, R | Cohort profile: UK Millennium Cohort Study (MCS). |  |  | International jof epidemiology | 10.1093/ije/dyu001 | 1719-1725 | 6 | 43 |
| 2011 | Crookston, BT | Impact of early and concurrent stunting on cognition | Childhood | Cognition | Matern Child Nutr | doi: 10.1111/j.1740-8709.2010.00255.x | 397-409 | 7 | 4 |
| 2010 | Crookston, BT | Children who recover from early stunting and children who are not stunted demonstrate similar levels of cognition | Childhood | Cognition | J Nutr | doi: 10.3945/jn.109.118927 | 1996-2001 | 140 | 11 |
| 2017 | Darling, AL | Association between maternal vitamin D status in pregnancy and neurodevelopmental outcomes in childhood: results from the Avon Longitudinal Study of Parents and Children (ALSPAC). | Childhood | Nutrition | The British j of nutrition | 10.1017/S0007114517001398 | 1682-1692 | 12 | 117 |
| 2018 | da Silva, ICM | Socioeconomic Inequalities Persist Despite Declining Stunting Prevalence in Low- and Middle-Income Countries | Childhood | growth | J Nutr | doi: 10.1093/jn/nxx050 | 254-58 | 148 | 2 |
| 2011 | Dewey, KG | Long-term consequences of stunting in early life. | Review | Growth | Maternal & child nutrition | 10.1111/j.1740-8709.2011.00349.x | May-18 | Suppl 3 | 7 Suppl 3 |
| 2018 | Donowitz, JR | Role of maternal health and infant inflammation in nutritional and neurodevelopmental outcomes of two-year-old Bangladeshi children. | Childhood | Nutrition | PLoS neglected tropical diseases | 10.1371/journal.pntd.0006363 | e0006363 | 5 | 12 |
| 2020 | Drago, F | Psychosocial and environmental determinants of child cognitive development in rural South Africa and Tanzania: findings from the MAL-ED cohort | Childhood | Cognition | BMC Public Health | doi: 10.1186/s12889-020-08598-5 | 505 | 20 | 1 |
| 2013 | Fall, CHD | Fetal malnutrition and long-term outcomes. | Review | Nutrition | Nestle Nutrition Institute workshop series | 10.1159/000348384 | Nov-25 |  | 74 |
| 2019 | Faye, CM | Factors associated with recovery from stunting among under-five children in two Nairobi informal settlements. | Childhood | Growth | PloS one | 10.1371/journal.pone.0215488 | e0215488 | 4 | 14 |
| 2014 | Fink, G | Childhood growth, schooling, and cognitive development: further evidence from the Young Lives study. | Adolescence | Growth | The American j of clinical nutrition | 10.3945/ajcn.113.080960 | 182-188 | 1 | 100 |
| 2013 | Galler, JR | Malnutrition in the first year of life and personality at age 40. | Adult | Nutrition | J of child psychology and psychiatry, and allied disciplines | 10.1111/jcpp.12066 | 911-919 | 8 | 54 |
| 2012 | Galler, JR | Socioeconomic outcomes in adults malnourished in the first year of life: a 40-year study. | Adult | Nutrition | Pediatrics | 10.1542/peds.2012-0073 | e1-7 | 1 | 130 |
| 2018 | Goncalves, H | Cohort Profile Update: The 1993 Pelotas (Brazil) Birth Cohort follow-up at 22 years | Childhood | Morbidity | Int J Epidemio | doi: 10.1093/ije/dyx249 | 1389-1390 | 47 | 5 |
| 2005 | Grajeda, R | The human capital study 2002-04: tracking, data collection, coverage, and attrition | Adult | nutrition | Food and Nutrition Bulletin | doi: 10.1177/15648265050262S103 | S15-24 | 26 | 2 Suppl 1 |
| 2013 | Gruszfeld, D | Early nutrition and health: short- and long-term outcomes. | Review | Nutrition | World review of nutrition and dietetics | 10.1159/000351482 | 32-39 |  | 108 |
| 1999 | Guerrant, DI | Association of early childhood diarrhea and cryptosporidiosis with impaired physical fitness and cognitive function four-seven years later in a poor urban community in northeast Brazil. | Childhood | Infection | The American j of tropical medicine and hygiene | 10.4269/ajtmh.1999.61.707 | 707-713 | 5 | 61 |
| 2014 | Hanieh, S | Maternal vitamin D status and infant outcomes in rural Vietnam: a prospective cohort study. | Childhood | Nutrition | PloS one | 10.1371/journal.pone.0099005 | e99005 | 6 | 9 |
| 2013 | Hoddinott, J | Adult consequences of growth failure in early childhood. | Adult | Nutrition | The American j of clinical nutrition | 10.3945/ajcn.113.064584 | 1170-1178 | 5 | 98 |
| 2008 | Hoddinott, J | Effect of a nutrition intervention during early childhood on economic productivity in Guatemalan adults | Childhood | nutrition | Lancet | doi: 10.1016/S0140-6736(08)60205-6 | 411-6 | 371 | 9610 |
| 2010 | Holme, A | The effects of breastfeeding on cognitive and neurological development of children at 9 years. | Childhood | Nutrition | Child: care, health and development | 10.1111/j.1365-2214.2009.01068.x | 583-590 | 4 | 36 |
| 2017 | Horta, BL | Associations of Linear Growth and Relative Weight Gain in Early Life with Human Capital at 30 Years of Age | Childhood | growth | J Pediatr | doi: 10.1016/j.jpeds.2016.12.020 | 85-91 | 182 | e3 |
| 2016 | Kaaya, S | Association of maternal depression and infant nutritional status among women living with HIV in Tanzania. | Childhood | Nutrition | Maternal & child nutrition | 10.1111/mcn.12154 | 603-613 | 3 | 12 |
| 2021 | Keats, EC | Effective interventions to address maternal and child malnutrition: an update of the evidence | Childhood | nutrition | Lancet child adolesc health | doi: 10.1016/S2352-4642(20)30274-1 | 367-384 | 5 | 5 |
| 2019 | Lai, JS | Maternal plasma vitamin B12 concentrations during pregnancy and infant cognitive outcomes at 2 years of age. | Childhood | Nutrition | The British j of nutrition | 10.1017/S0007114519000746 | 1303-1312 | 11 | 121 |
| 2019 | Lelijveld, N | Brain MRI and cognitive function seven years after surviving an episode of severe acute malnutrition in a cohort of Malawian children. | Childhood | Nutrition | Public health nutrition | 10.1017/S1368980018003282 | 1406-1414 | 8 | 22 |
| 2014 | Leroy, JL | Linear Growth Deficit Continues to Accumulate beyond the First 1000 Days in Low- and Middle-Income Countries: Global Evidence from 51 National Surveys | Childhood | growth | J Nutr | doi: 10.3945/jn.114.191981 | 1460-66 | 144 | 9 |
| 2019 | Leroy, JL | Perspective:What Does Stunting Really Mean? A Critical Review of the Evidence | Childhood | growth | Adv Nutr | doi: 10.1093/advances/nmy101 | 196-204 | 10 | 2 |
| 2019 | Li, C | Growth in early life and physical and intellectual development at school age: a cohort study. | Childhood | Growth | The British j of nutrition | 10.1017/S0007114519000060 | 866-876 | 8 | 121 |
| 2004 | Li, H | Relative importance of birth size and postnatal growth for women’s educational achievement | Childhood | growth | Early Hum Dev | doi: 10.1016/j.earlhumdev.2003.09.007 | 1-16 | 76 | 1 |
| 2006 | Lorntz, B | Early childhood diarrhea predicts impaired school performance. | Childhood | Morbidity | The Pediatric infectious disease j | 10.1097/01.inf.0000219524.64448.90 | 513-520 | 6 | 25 |
| 2014 | MacIntyre, J | Early childhood diarrhoeal diseases and cognition: are we missing the rest of the iceberg? | Childhood | Morbidity | Paediatrics and international child health | 10.1179/2046905514Y.0000000141 | 295-307 | 4 | 34 |
| 2008 | Manger, MS | A micronutrient-fortified seasoning powder reduces morbidity and improves short-term cognitive function, but has no effect on anthropometric measures in primary school children in northeast Thailand: a randomized controlled trial. | Childhood | Nutrition | The American j of clinical nutrition | 10.1093/ajcn/87.6.1715 | 1715-1722 | 6 | 87 |
| 2020 | Mansukoski, L | Four decades of socio-economic inequality and secular change in the pysical growth of Guatemalans | Childhood | socio-economic | Public Health Nutr | doi: 10.1017/S1368980019003239 | 1381-1391 | 23 | 8 |
| 2005 | Martorell, R | Rationale for a follow-up study focusing on economic productivity | Adult | nutrition | Food and Nutrition Bulletin | doi: 10.1177/15648265050262S102 | S5-14 | 26 | 2 Suppl 1 |
| 2013 | Mat&Child Nutrition Study Group | Maternal and child nutrition: building momentum for impact | Childhood | nutrition | Lancet | doi: 10.1016/S0140-6736(13)60988-5 | 372-75 | 382 | 9890 |
| 2007 | Neumann, CG | Meat supplementation improves growth, cognitive, and behavioral outcomes in Kenyan children. | Childhood | Nutrition | The J of nutrition | 10.1093/jn/137.4.1119 | 1119-1123 | 4 | 137 |
| 2019 | Neumann, D | A longitudinal study of antenatal and perinatal risk factors in early childhood cognition: Evidence from Growing Up in New Zealand. | Childhood | Cognition | Early human development | 10.1016/j.earlhumdev.2019.04.001 | 45-51 |  | 132 |
| 2010 | Oriá, RB | ApoE polymorphisms and diarrheal outcomes in Brazilian shanty town children. | Childhood | Morbidity | Brazilian j of medical and biological research = Revista brasileira de pesquisas medicas e biologicas | 10.1590/s0100-879x2010007500003 | 249-256 | 3 | 43 |
| 2005 | Oriá, RB | APOE4 protects the cognitive development in children with heavy diarrhea burdens in Northeast Brazil. | Childhood | Morbidity | Pediatric research | 10.1203/01.PDR.0000148719.82468.CA | 310-316 | 2 | 57 |
| 2005 | Patrick, PD | Limitations in verbal fluency following heavy burdens of early childhood diarrhea in Brazilian shantytown children. | Childhood | Morbidity | Child neuropsychology : a j on normal and abnormal development in childhood and adolescence | 10.1080/092970490911252 | 233-244 | 3 | 11 |
| 2016 | Pinkerton, R | Early Childhood Diarrhea Predicts Cognitive Delays in Later Childhood Independently of Malnutrition. | Childhood | Nutrition | The American j of tropical medicine and hygiene | 10.4269/ajtmh.16-0150 | 1004-1010 | 5 | 95 |
| 2018 | Ribe, IG | Determinants of early child development in rural Tanzania. | Childhood | Cognition | Child and adolescent psychiatry and mental health | 10.1186/s13034-018-0224-5 | 18 |  | 12 |
| 2004 | Richter, LM | Transition from Birth to Ten to Birth to Twenty: the South African cohort reaches 13 years of age | Adolescence | Socio-Demographic | Paediatr Perinat Epidemiol | doi: 10.1111/j.1365-3016.2004.00572.x. | 290-301 | 18 | 4 |
| 2018 | Rockers, PC | Two-year impact of community-based health screening and parenting groups on child development in Zambia: Follow-up to a cluster-randomized controlled trial. | Childhood | Intervention | PLoS medicine | 10.1371/journal.pmed.1002555 | e1002555 | 4 | 15 |
| 2008 | Santos, DN | Determinants of cognitive function in childhood: a cohort study in a middle income context. | Childhood | Cognition | BMC public health | 10.1186/1471-2458-8-202 | 202 |  | 8 |
| 2005 | Sachdev, HS | Anthropometric indicators of body composition in young adults: relation to size at birth and serial measurements of body mass index in childhood in the New Delhi birth cohort | Adult | growth | Am J Clin Nutrition | doi: 10.1093/ajcn.82.2.456 | 456-66 | 82 | 2 |
| 2017 | Sahar, N | Role of Family System, Positive Emotions and Resilience in Social Adjustment among Pakistani Adolescents | Adolescence | Socio-Demographic | J of Educational, Health and Community Psychology | (none) Vol 2(6):46-58 | 46-58 | 6 | 2 |
| 2018 | Scharf, RJ | Early childhood growth and cognitive outcomes: Findings from the MAL‐ED study | Childhood | growth/cognition | Matern Child Nutr | doi: 10.1111/mcn.12584 | e12584 | 14 | 3 |
| 2010 | Servili, C | Maternal common mental disorders and infant development in Ethiopia: the P-MaMiE Birth Cohort. | Childhood | Depression | BMC public health | 10.1186/1471-2458-10-693 | 693 |  | 10 |
| 2015 | Stafford, M | Childhood Environment and Mental Wellbeing at Age 60-64 Years: Prospective Evidence from the MRC National Survey of Health and Development. | Adult | Socio-Demographic | PloS one | 10.1371/journal.pone.0126683 | e0126683 | 6 | 10 |
| 2015 | Sudfeld, CR | Linear growth and child development in low- and middle-income countries: a meta-analysis | Childhood | growth | Pediatrics | doi: 10.1542/peds.2014-3111 | e1266-75 | 135 | 5 |
| 1996 | Pollitt, E | A Reconceptualization of the Effects of Undernutrition on Children's Biological, Psychosocial, and Behavioral Development | Childhood | nutrition | Society for Research in Child Development | (none) Vol 5(X): 1-32 | 1-32 | X | 5 |
| 2017 | Teivaanmäki,T | Height gain after two-years-of-age is associated with better cognitive capacity, measured with Raven's coloured matrices at 15-years-of-age in Malawi. | Adolescence | Growth | Maternal & child nutrition | 10.1111/mcn.12326 |  | 2 | 13 |
| 2018 | Undurraga, EA | Child stunting is associated with weaker human capital among native Amazonians. | Childhood | Growth | American j of human biology : the official j of the Human Biology Council | 10.1002/ajhb.23059 |  | 1 | 30 |
| 2020 | UNICEF, WHO, World Bank Group | Levels and Trends in Child Malnutrition | Childhood | nutrition | UNICEF, WHO, World Bank Group |  |  |  |  |
| 2007 | Vedhara, K | Relationship of early childhood illness with adult cortisol in the Barry Caerphilly Growth (BCG) cohort. | Adult | Morbidity | Psychoneuroendocrinology | 10.1016/j.psyneuen.2007.06.006 | 865-873 | 8-Oct | 32 |
| 2003 | Victora, CG | The Pelotas birth cohort study, RioGrande do Sul, Brazil, 1982-2001 | Childhood | Morbidity | Cadernos de Saude Publica | doi: 10.1590/s0102-311x2003000500003 | 1241-56 | 19 | 5 |
| 2008 | Victora, CG | Maternal and child undernutrition: consequences for adult health and human capital | Review | Nutrition | Lancet | 10.1016/s0140-6736(07)61692-4 | 340-57 | 9609 | 371 |
| 2021 | Victora, CG | Revisiting maternal and child undernutrition in low-income and middle-income countries: variable progress towards an unfinished agenda | Childhood | nutrition | Lancet | doi: 10.1016/S0140-6736(21)00394-9 | 1388-1399 | 397 | 10282 |
| 2018 | Visser, J | Community-based supplementary feeding for food insecure, vulnerable and malnourished populations - an overview of systematic reviews. | Review | Nutrition | The Cochrane database of systematic reviews | 10.1002/14651858.CD010578.pub2 | CD010578 | 11 | 11 |
| 1993 | Wachs, TD | Relations between nutrition and cognitive performance in Egyptian toddlers. | Childhood | Nutrition | Intelligence | 10.1016/0160-2896(93)90025-z | 151-172 | 2 | 17 |
| 2007 | Walker, SP | Child development: risk factors for adverse outcomes in developing countries | Childhood | nutrition/morbidity | Lancet | doi: 10.1016/S0140-6736(07)60076-2 | 145-57 | 369 | 9556 |
| 2015 | Zhu, P | Cord blood vitamin D and neurocognitive development are nonlinearly related in toddlers. | Childhood | Nutrition | The J of nutrition | 10.3945/jn.114.208801 | 1232-1238 | 6 | 145 |
|  |  |  |  |  |  |  |  |  |  |
| 1991 | (none) | Underlying and proximate determinants of child health: the Cebu Longitudinal Health and Nutrition Study | Childhood | Morbidity | Am J Epidemiology | (none). Vol 2(133):185-201 | 185-201 | 133 | 2 |
