# Supplementary material for: Examining the relationships between early childhood experiences and adolescent and young adult health status in a resource-limited population: A cohort study
Source: PLoS Med. 2021 Sep 28;18(9):e1003745. doi: 10.1371/journal.pmed.1003745 (PMC8478204; doi:10.1371/journal.pmed.1003745)
Supplement: S1 Appendix — (DOCX) [file pmed.1003745.s008.docx]

**Supplemental Information**

**S1 Appendix STROBE Checklist for Observational Studies**

|  | # | Recommendation | Reported in Section (Paragraph) | |  |
| --- | --- | --- | --- | --- | --- |
| **Title and abstract** | 1 | (*a*) Indicate the study’s design with a commonly used term in the title or the abstract | Title | |  |
|  |  | (*b*) Provide in the abstract an informative and balanced summary of what was done and what was found | Abstract  (Methods & Findings) | |  |
| Introduction | | | | |  |
| Background/rationale | 2 | Explain the scientific background and rationale for the investigation being reported | Introduction (3) | |  |
| Objectives | 3 | State specific objectives, including any prespecified hypotheses | Introduction (3) | |  |
| Methods | | | | |  |
| Study design | 4 | Present key elements of study design early in the paper | Introduction (3) | |  |
| Setting | 5 | Describe the setting, locations, and relevant dates, including periods of recruitment, exposure, follow-up, and data collection | Methods (3, 4) | |  |
| Participants | 6 | (*a*) *Cohort study*—Give the eligibility criteria, and the sources and methods of selection of participants. Describe methods of follow-up  *Case-control study*—Give the eligibility criteria, and the sources and methods of case ascertainment and control selection. Give the rationale for the choice of cases and controls  *Cross-sectional study*—Give the eligibility criteria, and the sources and methods of selection of participants | Methods (3-7) | |  |
|  |  | (*b*) *Cohort study*—For matched studies, give matching criteria and number of exposed and unexposed  *Case-control study*—For matched studies, give matching criteria and the number of controls per case | N/A | |  |
| Variables | 7 | Clearly define all outcomes, exposures, predictors, potential confounders, and effect modifiers. Give diagnostic criteria, if applicable | Methods  (3, 5-7) | |  |
| Data sources/ measurement | 8* | For each variable of interest, give sources of data and details of methods of assessment (measurement). Describe comparability of assessment methods if there is more than one group | Methods  (5-7) | |  |
| Bias | 9 | Describe any efforts to address potential sources of bias | Methods (4)  Results (1) | |  |
| Study size | 10 | Explain how the study size was arrived at | Methods (3,4) | |  |
| Quantitative variables | 11 | Explain how quantitative variables were handled in the analyses. If applicable, describe which groupings were chosen and why | Methods  (3, 5-7) | |  |
| Statistical methods | 12 | (*a*) Describe all statistical methods, including those used to control for confounding | Methods (8) | |  |
|  |  | (*b*) Describe any methods used to examine subgroups and interactions | Methods (8) | |  |
|  |  | (*c*) Explain how missing data were addressed | Methods (7)  Figure 1,2 | |  |
|  |  | (*d*) *Cohort study*—If applicable, explain how loss to follow-up was addressed  *Case-control study*—If applicable, explain how matching of cases and controls was addressed  *Cross-sectional study*—If applicable, describe analytical methods taking account of sampling strategy | Methods (7)  Fig 1,2 | |  |
|  |  | (*e*) Describe any sensitivity analyses | Methods (8) | |  |
| **Results** |  |  |  | |  |
| Participants | 13* | (a) Report numbers of individuals at each stage of study—e.g. numbers potentially eligible, examined for eligibility, confirmed eligible, included in the study, completing follow-up, and analysed | Fig 1 | |  |
|  |  | (b) Give reasons for non-participation at each stage | Fig 1 | |  |
|  |  | (c) Consider use of a flow diagram | Fig 1 | |  |
| Descriptive data | 14* | (a) Give characteristics of study participants (eg demographic, clinical, social) and information on exposures and potential confounders | Results (1,2)  Table 1 | |  |
|  |  | (b) Indicate number of participants with missing data for each variable of interest | Fig 1 | |  |
|  |  | (c) *Cohort study*—Summarise follow-up time (eg, average and total amount) | Reference 9 | |  |
| Outcome data | 15* | *Cohort study*—Report numbers of outcome events or summary measures over time | Fig 2, 3, 4 | |  |
|  |  | *Case-control study—*Report numbers in each exposure category, or summary measures of exposure | N/A | |  |
|  |  | *Cross-sectional study—*Report numbers of outcome events or summary measures | N/A | |  |
| Main results | 16 | (*a*) Give unadjusted estimates and, if applicable, confounder-adjusted estimates and their precision (eg, 95% confidence interval). Make clear which confounders were adjusted for and why they were included | Results (1-6)  Fig 2, 3, 4 | |  |
|  |  | (*b*) Report category boundaries when continuous variables were categorized | Methods (7)  Table 1 | |  |
|  |  | (*c*) If relevant, consider translating estimates of relative risk into absolute risk for a meaningful time period | N/A | |  |
| Other analyses | 17 | Report other analyses done—eg analyses of subgroups and interactions, and sensitivity analyses | | Results (3-5),  Fig 3, 4  Tables S2, S3, S4 & S5 | |
| **Discussion** |  |  | |  | |
| Key results | 18 | Summarise key results with reference to study objectives | | Discussion (2) | |
| Limitations | 19 | Discuss limitations of the study, taking into account sources of potential bias or imprecision. Discuss both direction and magnitude of any potential bias | | Discussion (11) | |
| Interpretation | 20 | Give a cautious overall interpretation of results considering objectives, limitations, multiplicity of analyses, results from similar studies, and other relevant evidence | | Discussion (13) | |
| Generalisability | 21 | Discuss the generalisability (external validity) of the study results | | Discussion (2,5,8) | |
| Other information |  |  |  |  |  |
| Funding | 22 | Give the source of funding and the role of the funders for the present study and, if applicable, for the original study on which the present article is based | | Funding statement | |
