# Supplementary material for: Examining the relationships between early childhood experiences and adolescent and young adult health status in a resource-limited population: A cohort study
Source: PLoS Med. 2021 Sep 28;18(9):e1003745. doi: 10.1371/journal.pmed.1003745 (PMC8478204; doi:10.1371/journal.pmed.1003745)
Supplement: S2 Appendix — (DOCX) [file pmed.1003745.s009.docx]

**S2 Appendix**

**Standard Operating Procedure *Young Adult Follow-Up (YAFU) Form***

***Component of Oshikhandass Water and Sanitation, Health and Hygiene Interventions (WSHHI) Study***

I. Purpose:
To collect current health and socioeconomic information on the cohort of participants in the 1989-1996 Oshikkhandass Diarrhea and Pneumonia study. Information collected will include anthropometry, general health and family history, history of hospitalization and acute illnesses, educational achievement, employment status, migration and travel patterns, and community involvement.

II. Materials:
Young Adult Follow-Up (YAFU) Form, pen, standard measuring tape and straight edge for leveling off OR (ideally) portable stadiometer (cm, HM200P PortStad Portable Stadiometer), battery or solar-powered weight scale (kg, Seca Digital), blood pressure gauge (mm/Hg, Yamasu Model 600, Japan), stethoscope, and Raven’s Standard Progressive and Colored Progressive Matrices booklets (PsychCorp).

III. Methods:
A. A Research Coordinator trained in using the YAFU survey will administer this form. This form will be administered to every participant in the 1989-1996 cohort. Whenever possible, this form will be administered in conjunction with the Raven’s Progressive Matrices (RPM) assessment (directions to be found in separate Standard Operating Procedures manual). The YAFU and RPM should not be administered at separate times. Only when absolutely necessary, the RPM may be administered at a different time than the YAFU.

B. *Above the boxes* at the top-right of each page, labeled for Participant Identification Number (new ID from the current WSHHI study), please write the 7-character alphanumeric Individual Identification Number (old ID from the 1989-1996 study).

Explanations for each question in the form will follow, but for identification purposes, be sure to enter the Individual ID (old ID, 7-character, from the 1989-1996 study), Participant ID (new ID, 8-character, from the current WSHHI study) if available, interviewer’s name and code, the respondent’s name (both reported during interview and previously recorded in the Child Database), and the day, month, and year the form is first being filled (Format: DD/MMM/YY). Please note that all abbreviations should be capitalized*.* Year abbreviation will be the last two digits of the calendar year (Ex. January 20, 2012 will become 20 JAN 12). Month abbreviations are as follows:

| January = JAN | February = FEB |
| --- | --- |
| March = MAR | April = APR |
| May = MAY | June = JUN |
| July = JUL | August = AUG |
| September = SEP | October = OCT |
| November = NOV | December = DEC |

C. Each YAFU participant should be contacted at least one day prior to the appointment when the form will be administered. The list of participants from the 1989-1996 cohort to be interviewed is available in the Oshikhandass Child Database, which is being updated with contact information and addresses of current participants in the YAFU Registry. Only the participant specified in the Child Database may be interviewed – family members or friends may not provide information in the participant’s absence. Records such as education certificates may, however, be received from family members.

D. At the time this protocol is being finalized, there are approximately 968 participants still living in Oshikhandass, 439 outside of Oshikhandass, 338 who were not found, and 121 who have died. Since many YAFU participants have migrated outside of the project site in Oshikhandass, rare cases (such as if the participant is living in a foreign country or an otherwise inaccessible area in-country) are expected to arise where YAFU participants are unable to be visited by a research team member for face-to-face interview. In these cases the YAFU form may be administered on the telephone. In these cases, however, it will not be possible to administer the Raven’s Matrices assessment, nor record anthropometry data.

E. Any YAFU forms filled which deviated from the normal course will be marked as such. “Telephone” will be written in the left-hand corner of the form when the form was filled by telephonic interview.

F. When identifying the YAFU participant, some of their names have changed since the name recorded at registration (1989-1996). It may help to identify individuals according to the names of other family members, recorded date of birth, or even who their neighbors were.

**Explanation and Rationale (Modified YAFU Form)**

The YAFU form was modified in October 2012 for implementation in the field in November 2012. Training on the form was completed in October 2012, as the form was developed alongside piloting exercises from July-September 2012. The YAFU serves to provide current health and socioeconomic information on the cohort of participants in the 1989-1996 study in Oshikhandass. The YAFU will also help understand the social aspects of illness, suffering, and development in the community, as well as potential areas for future research.

**The following is an explanation of each question.**

*Pre-coded answer choices should be circled. Where “Other (specify)” is an option, no code should be written in to correspond to “Other,” but “Other” should be circled and the desired response should be written out in the coding section on the left-hand side of the form.*

*All the questions herein are open-ended, except Yes/No-type questions. The interviewer should not prompt the respondent for any specific responses nor read off the pre-coded answer options. The interviewer may probe or make a suggestion to the respondent if the respondent’s initial answer(s) seem to fall in the category of one of the pre-coded answer options, but is not explicitly worded as such. If prompting is required to give the respondent an example answer, all possible answer choices should be shared.*

*There are many questions in the survey for which the respondent may have multiple answers. Please mark all answers provided by the respondent. Additional answer choices should be clearly written in next to the pre-written answer options.*

**Section 1 – Basic Information:** This section is intended to record basic identification information and to monitor whether or not a Raven’s Progressive Matrices assessment was completed. It is *absolutely vital* that this section be completely and accurately written in, since it involves matching identification records across multiple databases.

**1:** Participant ID (New ID):

- In the 8 boxes to the top right of the form, please fill in one character in each box for all of the 8 characters of the participant’s “New ID.” This is the identification number that was assigned to every individual in Oshikhandass when remapping the village in 2011. The New ID can be found in the Current Household Member Database.
- It is important to note that not all individuals may have a Participant ID, especially if they migrated away from Oshikhandass before the most recent WSHHI mapping.

**2:** Individual ID (Old ID):

- In the 7 boxes in Question 2, please fill in one character in each box for all of the 7 characters of the participants “Old ID.” This is the identification number that was assigned to every individual in Oshikhandass during the 1989-1996 study. The Old ID can be found in the Child Database 1989-1996.
- When scheduling and conducting YAFUs, it must be confirmed that all information – name, date of birth, mother and father’s names, etc. – matches between the Participant ID (New ID) and the Individual ID (Old ID).
- Since we are working off of the 1989-1996 Child Database as the follow-up tool, the Individual ID is the primary identifier.

**3:** Date of Visit

- Date of visit is the date in which the respondent is being interviewed in follow- up. (Format: DD/MMM/YY).

**4**: Interviewer

- Research Coordinator/ Field Supervisor who interviews for follow-up purpose will mention his/her code.
- **Codes:** 01 = AJ; 02 = FS; 03 = WS; 04 = EB

**5**: Name of Respondent (Official/Legal?)

- The Official or legal name of the respondent is that particular name mentioned in School/College/University documents or in Computerized National Identity Card (CNIC).
- **Note:** The interviewer should be sure that this is in fact the “Official” name, which is recorded in the respondent’s documents.

**6:** Recorded Name (Old)

- The recorded name of the participant mentioned in the previous study in the 1989-1996 study in Oshikhandass. The Recorded name can be found in the Child Database 198691996.

**7:** Nickname

- Here, record if the respondent reports a commonly used name other than their Official/Legal name. Otherwise, this may be coded as 97.

**8:** Mother’s Name

- Name of the mother of the participant. Ask for the exact spelling and the name which the mother is most commonly known by (not necessarily the name written in documents).
- **Note:** Though the mother’s name which was originally recorded in the old Oshikhandass study is available in the Child Database, it is best to record the newer/more commonly used name in order to avoid confusion going forward.

**9:** Father’s Name

- Name of the father of the participant. Ask for the exact spelling and the name which the father is mostly commonly known by (not necessarily the name written in documents).
- **Note:** Though the father’s name which was originally recorded in the old Oshikhandass study is available in the 1989-1996 Database, it is best to record the newer/more commonly used name in order to avoid confusion going forward.

**10:** Reported DOB

- In the spaces provided, write the respondent’s Official Date of Birth, mentioned in the School/College/University official or legal documents or in Computerized National Identity Card (CNIC) (Format: DD/MMM/YY).
- **Important Note:** Also ask, “Is your Official/Reported Date of Birth the same as your Actual Date of Birth?” This is because individuals often change their date of birth for documentation purposes, usually in the way of decreasing one’s reported age.
- If the Actual Date of Birth is different than the Official/Reported Date of Birth, then write the Actual in parentheses beside the Reported Date of Birth.

**10a:** Recorded DOB

- The recorded DOB of the participant mentioned in the previous 1989-1996 study in Oshikhandass. The Recorded DOB can be found in the Child Database 1989-1996 (Format: DD/MMM/YY).

**11:** Age (from Reported DOB)

- Calculated time duration from date of birth to the reported date, in years and months.

**12:** Address

- Address refers to the present address where the respondent is currently living.
- Please get as much detailed info about address as possible, not just the city.

**13:** Contact Number

- The cell phone or land line number, which may be used to contact the participant.

**14:** Email

- The participant’s email address.

**15:** Raven’s Matrices administered?

- Has the interviewee completed the Raven’s Progressive Matrices test?
- **Codes:** (01) Yes, (02) No

**Section 2 – Anthropometry:** This section aims to measure the physical body statistics of the follows-up children. The measurement includes body height, weight, waist girth, and blood pressure.

**1:** Height:

- The height will be measured using a portable stadiometer, in centimeters, to one tenth centimeter significant figure. (Format: xxx. x cm)
- **Note:** Be sure the individual takes his/her shoes/sandals off, his/her feet are placed together. The heels, buttocks, and shoulder blades should be touching the height board (or wall, if tape measure being used), and the individual should generally be standing straight.
- In the case that the stadiometer cannot be used, and a measuring tape must be used instead, the same rules apply for making sure the participant is standing straight. Additionally, use a straight edge to level off the participant’s measurement at his/her head, making sure to angle the straight edge exactly perpendicular to the top of his/her head. Mark the height on the wall with a pencil and use the measuring tape to measure.

Standing Height Position^[[1]](#footnote-1)^

**2:** Waist Girth:

- Waist girth will be measured using a standard measuring tape, in centimeters, to one tenth centimeter significant figure. (Format: xxx .x cm)
- **Note:** Measurement should be taken while the individual is in standing position. The measuring tape should be placed around the top of the pelvic bone, where the bone meets the waist, and generally near the waist circumference around the belly-button (though this is not true with all individuals).
- Place the end points of the measuring tape toward one side of the individual, which facilitates ease of reading. Placing one finger in between the individual’s waist and the tape, hold the tape in place, letting the tips overlap, and see where the beginning of the tape meets the tape again. Tell the individual to breathe in, and then breathe out, to ensure the tape is in the optimal position. Take the reading when the individual has exhaled and relaxed.

**Left:** The + marks the position to place the tape. **Right:** This is an alternate view of how the tap should be placed.^[[2]](#footnote-2)^

**3:** Weight:

- Weight will be measured using a Seca Digital weight scale (solar-powered used in Gilgit, battery-powered down-country), in kilograms, to one tenth kilogram significant figure. (Format: xxx.x kg)
- **Note:** Be sure to place the weight scale on a flat surface, such as tile or cement. Do not place the weight scale on a carpet, as this provides a slightly defective reading. Before measuring weight, ask the participant to take off his/her shoes and empty his/her pockets.
- After turning the weight scale on, make sure the scale “zeroes” (reads “0.0”) before the participant steps onto the scale. The individual should be standing fully on the scale, and should not step off until the scale fixes on a given weight, and the number flashes on the screen.

**4:** Blood Pressure:

- Blood pressure will be measured on the right arm using a mercury blood pressure gauge and stethoscope, recording both systolic and diastolic measures. (Format: mm Hg/mm Hg).

Blood Pressure Gauge Placement and Measurement^[[3]](#footnote-3)^


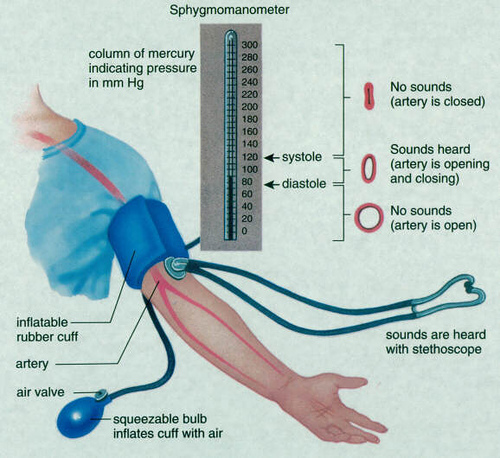


**Section 3 - Health and Geography Questions:** This section aims to measure health-related information such as general health status, specific health problems encountered, geographic location of the individual and access to health facilities in the region.

**1:** How would you characterize your health in general?

- **Codes**: (01) Excellent, (02) Good, (03) Satisfactory, (04) Poor, (05) Very poor

**1a:** If poor/very poor, explore why?

- If the individual characterized his/her health as poor or very poor, ask the individual to explain why and record the response.

**2:** How would you characterize your health as a child (ages 5-15)?

- **Codes:** (01) Excellent, (02) Good, (03) Satisfactory, (04) Poor, (05) Very poor

**2a:** If poor/very poor, explore why?

- If the individual characterized his/her health as poor or very poor, ask the individual to explain why and record the response.

**3:** Have you had any specific health problems in your life?

- **Codes:** (01) Asthma, (02) Recurrent pneumonia, (03) Repeated ear infections, (04) Recurrent Diarrhea, (05) Worms, (06) Concerns about blood pressure: High/Low, (07) Major Accident, (08) Broken bones, (09) Surgery, (10) Depression, or other emotional problems, Other (specify):
- **Note:** Circle all answers given. If complicated comments, write in section provided in Question 4b.

**4:** Have you ever been hospitalized?

- **Codes:** (01) Yes, (02) No
- **Skip:** If “No” to Question 4, skip to Question 4b.

**4a:** If yes (you have been hospitalized), where and for what?

- Indicate the date the individual was admitted into the hospital, the date the individual was discharged, the name and place of the hospital, the reason(s) for which the individual was hospitalized, and the outcome. Put each instance when the individual was hospitalized in a new row.
- **Note:** Since many participants don’t remember exact dates of hospitalization/discharge, estimation is OK. Estimate to whatever accuracy (day, week, month, or year) that the respondent remembers clearly. Since exact dates won’t be known, *do* ask for a clear answer on the number of days and nights hospitalized and record this – determining duration of stay in the hospital is the main intention of this question.
- **Outcome Codes:** (1) Cured, (2) Treated but not cured, (3) Left against medical advice (LAMA)

| S/N | Date of hospitalization | Date of discharge | Name of hospital (place) | For what problem (more than one response possible) | Outcome(s)  1: cured  2: treated but not cured  3: LAMA |
| --- | --- | --- | --- | --- | --- |
| A |  |  |  |  |  |
| B |  |  |  |  |  |
| C |  |  |  |  |  |
| D |  |  |  |  |  |

**4b:** Other comments about medical history, whether or not hospitalized:------------------------------------------------------------------------------------------------------------------------------------------------------------------------------------------------------------------------------------------------------------------------

**5:** Do you feel like you have access to adequate healthcare in Oshikhandass?

- **Codes:** (01) Yes, (02) No

**5a:** If no, how far (in km) do (or would) you or your family travel for healthcare?

- **Codes:** No codes. Write the response in kilometers.

**5b:** Which cities have you traveled to (especially) for healthcare?

- **Codes**: (01) Gilgit, (02) Islamabad, (03) Lahore, (04) Karachi, (05) Other (specify):

**6:** Do you still live in Oshikhandass?

- **Codes:** (01) Yes, (02) No
- **Skip:** If “Yes” to Question 6, skip to Section 4
- **7:** Where do you live now? **No codes:** Write the name of the city/town in which the respondent is currently living.

**7a:** How long have you lived in your new city?

**Codes:** No codes. Write how long the individual has lived in the current city, in years and months.

**8:** At what age did you initially move outside Oshikhandass?

- **Codes:** No codes. Write the individual’s age in years and months.

**8a:** Reason(s) for initially moving (more than one response possible)?

- Ask the individual why he/she initially moved away from Oshikhandass. Multiple answers are possible.
- **Codes:**(01) School, (02) Work, (03) Married, (04) Family Reasons, Other (specify):________

**9:** Do you feel like you have access to adequate healthcare in your city?

- **Codes:** (01) Yes, (02) No
- **Skip:** If “Yes” to Question 9, skip to Section 4, Question 1.

**9a:** If no, how far (in km) do you or family travel for healthcare?

- **Codes:** No codes. Write the response in km

**Section 4 - General Questions:** This section provides information about marital status, duration of marriage, number of children and current occupational status of the respondent.

**1:** Are you married?

- **Codes:** (01) Yes, (02) No
- **Skip:** If “No” to Question 1, skip to Question 3.

**1a:** If yes, for how many years?

- If the individual is currently married, ask how long he/she has been married.
- **Codes:** No codes. Write the response in years and months

**Skip:** If not married 9 months or more, skip to Question 3.

**2:** Do you have children?

- **Codes:** (01) Yes, (02) No
- **Note:** If the individual has children, fill in the table below with one child per row. Indicate the gender of the child and if the child is attending school. If the child is currently attending school, indicate which school in the second column from the right. If the child is not attending school, indicate why in the right most column.
- **Codes for why the child is not attending school:** (01) Too young, (02) could not afford, (03) other (specify).

|  | Gender: (01) Male (02) Female | Attending school? (01) Yes (02) No | If attending school, which school: | If not attending reason(s)?(01) Too young (02) Could not afford (03) Other (specify) |
| --- | --- | --- | --- | --- |
| A |  |  |  |  |
| B |  |  |  |  |
| C |  |  |  |  |
| D |  |  |  |  |

**3:** What are you doing now?

- Ask the individual about his/her current job and educational status.
- **Codes:**(01) Full-time student, (02) Part-time student, (03) Full-time student & employed, (04) Part-time student & employed, (05) Completed studies & employed, (06) Completed studies & unemployed, (07) Not in labor force & doing unpaid family work, (08) Ongoing studies intended & break in education
- **Note:** Individuals who are usually earning but now out of work who have the desire and ability to work, who are seeking and available for work, are unemployed.
- If someone, usually a female, has been married and living at home, not doing any other work, then the answer to this question is (07) Not in labor force & doing unpaid family work

**3a:** How long has this been your status?

- Ask the individual how long he/she has had the status mentioned in Q3.
- **Codes:** No codes. Record response in years and months.
- **Note:** This question is asking how long the participant has been doing whatever their response was in Q3 above. For example, if the individual has only attended school his/her entire life (01) Full-time student, then the purpose of this question is to ask, “How many years has it taken to get to this point in your schooling?” If (04) Part-time student and employed for Q3, then you should ask them how long they have been going to school part-time and working at the same time. Similarly, if the answer is (07), you should ask them for how long they have not been in the labor force and/or doing unpaid family work.
- **Skip:** If student who is not working (answer options 01-02), skip to Section 6; otherwise, administer Section 5.
  - **Important Note**: On the form, the skip-pattern for this question reads “If student who is not working (answer options 01-04), skip to Section 6…” This is INCORRECT. It should be that if someone ONLY is a student, then skip to Section 6. i.e. If someone is working at all (i.e. but is unemployed), *or* has worked in the past for some significant period (3 months or more), in some capacity other than community volunteering, then Section 5 must be administered.

**Section 5- Employment Questions:** This section is developed to measure the employment status of the individuals and determine their current employment/ unemployment status and how they earn their livelihood.

**1:** Are you currently employed or have you been employed in the last 6 months?

- **Codes:**(01) Currently employed, (02) Employed within last 6 months, but not currently employed, (03) Not employed in last 6 months
- **Skip:** If “Not employed in last 6 months”(03), skip to Question 3. If “Currently employed” (01), skip to Question 5.

**2:** If you were employed in the last 6 months, but are not employed now, why did you end your job?

- **Codes:**(01) Fired, (02) Laid off, (03) Unhappy at job (left by choice), (04) Want to switch jobs, Other (specify):­­­­___________

**3:** If not employed, are you available for work?

- **Codes:** (01) Yes, (02) No
- **Note:** A person who is available for work is one who is willing and able to work.

**4:** If not employed, are you looking for work?

- **Codes:** (01) Yes, (02) No
- **Skip:** If “No” to Question 4, skip to Question 5.
  - **Important Note:** On the form, the skip-pattern for this question reads “If “No” to Question 4, skip to Question 12.” This is INCORRECT.

**4a:** If yes (you are not employed and have been looking for work), how long have you been looking for?

- **Codes:** No codes. Write response in years and months.

**5:** What is/was your field of work?

- **Codes:** No codes. Write response provided by participant. Coding will be done at later time.
- **Note:** If the participant is currently employed, ask what field he/she is currently working in. If the participant is unemployed, ask what field he/she was previously working in. If the individual ask what field they intend to go into.

**Important Note on Questions 6-11:** If never employed (i.e. but looking for work), fill “97” for “Not Applicable” for Questions 6-11. If employed at some point, then each of these questions are effectively modified to read what “was” (rather than “is”) your organization’s name, type of organization, income, etc.

**6:** What is (or was) your business or organization’s name?

- **Codes:** No codes. Write response provided by participant.

**6a:** What type of organization is (or was) this?

- **Codes:** (01) Service/organization, (02) Business, (03) Personal or family business, Other (specify):_____________

**7:** What is (or was) your job title?

- **Codes:** No codes. Write response provided by participant. Coding will be done at later time.

**8:** What is (or was) your monthly income?

- **Codes:** No codes. Write response provided by participant in rupees.

**9:** How many hours per week are you working (or did you work)?

- **Codes:** No codes. Write response provided by participant in hours per week.
- **Note:** This question may require probing to find out how many hours per day an individual works, then how many days per week, and finally multiplying to determine hours per week.

**10:** Are you looking for more work?

- **Codes:** (01) Yes, (02) No
- **Note:** An individual who is looking for more work is someone who is either a) unemployed and would like to be employed or someone who is b) currently employed but would like to work more hours. If unemployed, the answers to Question 10 will be the same as that to Question 4.

**11:** Are you currently satisfied with your employment status?

- **Codes:** (01) Yes, (02) No

**12:** Do you hope to enter a different field of employment?

- **Codes:** (01) Yes, (02) No
- **Skip:** If “No” to Question 12, skip to Question 13.

**12a:** If yes, what field?

- If the individual indicated that he/she would like to enter a different field of employment (answered (01) “yes” to Q12, ask the individual what field he/she would like to enter.

**13:** Do you have any other ways of supporting yourself financially other than in Question 1 above (e.g. other personal business, loan, investment, rental revenue)?

- **Codes:** (01) Yes, (02) No
- **Skip:** If “No” to Question 13, skip to Question 14.
- **Note:** Ask the individual if he/she is support himself/herself by means in addition to that mentioned in Q1.

**13a**: If yes, describe:

- If the participant has other means of supporting himself/herself as indicated in Question 13, ask him/her to describe these means. Write the participant’s response.

**14:** Do you have any other unpaid work you do?

- **Codes:** (01) Yes, (02) No
- **Skip:** If “No” to Question 14, skip to Question 15.
- **Note:** If someone is volunteering for a community organization, this will be covered in Section 8, Question 1. However, if someone is serving as an intern or apprentice somewhere, or specifically mentions doing work for the household, such as farming or other household tasks, then this should be recorded in this question.

**14a:** If yes, describe:

- If the participant indicates in Q14 that he/she does unpaid work, ask him/her to explain what time of work this is.

**15:** Do you have any financial debt?

- **Codes:** (01) Yes, (02) No
- **Skip:** If “No” to Question 15, skip to Section 6, Question 1.

**15a:** If yes, how much?

- If the participant indicated in Q15 that he/she has financial debt, ask him/her to indicate how much debt in rupees.

**Section 6 –Education Questions:** This section aims to determine educational status, literacy rate, reasons for illiteracy, and desire for further education.

**Note:** If Never Enrolled, then skip to Question 6 (page 12 of form).

**1:** Did you ever skip any classes?

- **Codes:** (01) Yes, (02) No
- **Skip:** If “No” to Question 1, skip to Question 2.

**1a:** If yes, specify classes (e.g. 1^st^, 2^nd^, 3^rd^, etc.):

- **Codes:** skip classes name
- **Note:** If the participant skipped class(es), indicate which class(es).

**2:** Did you ever have to repeat any classes?

- **Codes:** (01) Yes, (02) No
- **Skip:** If “No” to Question 2, skip to Question 3.

**2a:** If yes, specify classes and number of times:

- If the individual had to repeat any class, write each class that was repeated on a new row in the table below. Indicate which class it was and the number of times it was repeated.

|  | Class Repeated (e.g. Prep, 1^st^, 2^nd^, etc.) | Number of Times Repeated (e.g. 1, 2, 3, etc.) |
| --- | --- | --- |
| A |  |  |
| B |  |  |
| C |  |  |
| D |  |  |

**3:** Did you ever have to take any supplementary exams?

- **Codes:** (01) Yes, (02) No
- **Skip:** If “No” to Question 3, skip to Question 4.

**3a:** If yes, specify years and subjects:

-If the participant took supplementary exams, write each exam on a new row of the table below. Indicate the subject of the exam, the year of supplementary and the number of times it was taken.

|  | Subject of Exam (e.g. English, Physics, Islamiat, etc.) | Year of Supplementary (e.g. Intermediate year 2, University year 1, etc | Number of times taken |
| --- | --- | --- | --- |
| A |  |  |  |
| B |  |  |  |
| C |  |  |  |

**4:** Did you ever take an English class?

- **Codes:** (01) Yes, (02) No
- **Skip:** If “No” to Question 4, skip to Question 5 (Education Table).
- **Note:** This question is asking if the participant took any class to learn English, i.e. English as a subject in school. This is not referring to English medium classes or separate special tuition.

**4a:** If yes, from which grade level (e.g. Prep, 1, 2, 3, etc.)?

- **Code:** No codes. Indicate the class name.
- **Note:** Multiple grade levels are possible. Please list all responses provided.

**Please specify where you attended school from Primary through University or Professional Training, up through your highest level of education, including degrees and certificates obtained:**

**5: Use the following codes for the table on the next three pages:**

- **Note:** Where possible, verify the information provided by the participant by viewing educational certificates and records.

1. Write the name of the institution e.g. Karachi University; Aga Khan Diamond Jubilee School, Oshikhandass

Institution Type: e.g. Government, AKES, private, madrasa, or other (specify)

Years Attended: e.g. 2010-2012; 2010-IP for schooling still in progress

Degree Type: e.g. MPhil for University; BSc for College; FSc for Intermediate; Certificate for Matric, Middle School, or Primary School

Area of Study/Subject: e.g. Chemistry (for Bachelor’s or above); Science or Arts (for Matric); Pre-Medicine or Pre-Engineering, etc. (for Intermediate)

**Note:** “Area of Study” is not applicable for Primary or Middle School. This can be left blank there. Please write the specific field of study, not general “Bachelor’s,” etc.

Board: e.g. KIU, ­Federal – Islamabad, Karachi, Punjab, Aga Khan, or other (specify)

Final Grade: e.g. A or B; record percentages for degree subject, Urdu, English, and Math if known

**Note:** For the “Final Grade” column, it is most important that you get reliable information. The most important info is for Matric/High School. Other grades are not necessary. If the respondent can reliably remember Matric grades, record this. However, leave this blank if he/she clearly does not remember.

Wherever you note down any Final Grade, you should write in the farthest left column (where class/grade level is listed) whether or not the degree and percentages were directly observed. Write OBSERVED or NOT OBSERVED/RECALL. This is why it is important to make appointments with participants beforehand and tell them to bring all their educational documents.

1. Reason for Change From Previous School (if any change): (01) Better school, (02) More affordable, (03) Closer to home, (04) Family moved, (05) Education not offered beyond this level at previous school, (06) Personal preference (e.g. for friends) Other (specify)

|  | **1** | **2** | **3** | **4** | **5** | **6** | **7** | **8** |
| --- | --- | --- | --- | --- | --- | --- | --- | --- |
| Institution Level | Institution Name | Institution Type | Years Attended | Degree Type | Area of Study | Board | Final Grade | School Change |
| Professional Training and Other Certificates / Diplomas (LHW/PTC/CT/Montessori/madrasa/etc) |  |  |  |  |  |  |  |  |
| University for Master’s or above (MA/MSc/MBA/MPhil/PhD/etc) |  |  |  |  |  |  |  |  |
| College or University for Bachelor’s (BA/BSc/BComm/etc) |  |  |  |  |  |  |  |  |
| Intermediate (FA/FSc/IComm) |  |  |  |  |  |  |  |  |
| Matric/High School (9-10) |  |  |  |  |  |  |  |  |
| Middle School (6-8) |  |  |  |  |  |  |  |  |
| Primary (prep-5) |  |  |  |  |  |  |  |  |
| Has the certificate or degree for the highest level of education completed been observed? | | | | | **Codes:** (01) Yes (02) No(03) In Progress | | | |

- **Comments (e.g. if break taken in education, awards or honors received, or other hardships, please take note): ___________________________________________________________________________________________________________________________________________**
- **Note: Ask all questions, i.e. If there were other difficulties in education, if there was a break in education (and why), and whether awards or honors were received.**

**6:** At what class/grade level did you stop your education?

- **Codes:** (00) Nursery/prep, (98) Never enrolled, (99) Ongoing education; Otherwise, write grade level.
- **Skip:** If “Ongoing education” (99), skip to Question 8. If stopped education (00, 98, or grade level entered), then fill Questions 7-8, skip Question 9, and then skip to Section 7, Question 1.
- **Note:** Here, ask probing, “Do you intend on further pursuing education?” This answer will guide for Questions 7-9.

**7:** At what age did you stop your education?

- **Codes:** (98) Never enrolled, (99) Ongoing education; Otherwise, write age in years.
- **7a:** Once you stopped your education, why did you not pursue further education? **Codes:** (01) Was not interested, (02) Could not afford, (03) Needed to earn for family, (04) Physical disability, (05) Illness, Other (specify)
- **Note:** If Never Enrolled, code 97

**8:** Which education level has been completed or is in progress?

- **Codes:** (01)Primary, (02)Middle, (03)High school/Matric, (04) FA/FSc/FComm, (05) BA/BSc/BComm, (06) MA/MSc/MBA, (07) MPhil/PhD, (08) (Professional training) (LHW/PTC/CT/Montessori/madrasa/etc)
- **Note**: Mark all that apply.

**9:** If ongoing education, which level of education do you intend to study toward?

- **Codes:** (01)Primary, (02)Middle, (03)High school/Matric, (04) FA/FSc/FComm, (05) BA/BSc/BComm, (06) MA/MSc/MBA, (07) MPhil/PhD, (08) (Professional training) (LHW/PTC/CT/Montessori/madrasa/etc)

**Section 7- Travel History: -** This section serves to measure worldly exposure, as a function of travel, cities visited, and reasons for travel**.**

**1:** Have you been to any of these cities/areas?

- **Note:** Ask the participant if he/she has been to each of these cities/areas. If he/she has been to a city/area, indicate the number of times (for Gilgit, indicate the number of times per month, on average, i.e. when they were living in Oshikhandass if they have since moved away) and the reason for the visit.
- If other cities also visited, manually enter the name of the city, the number of times visited, and reasons (as pre-figured spaces are not provided on the form).
- **Frequent Responses:** To be coded; Family/relatives/wedding, Shopping, Medical/Hospital, Picnic/camping, Job, School, or Traveling/in transit (e.g. if stopped in Rawalpindi/Islamabad on way to another city)

| **1a** | Gilgit | (01) Yes (02) No | No. times per month | Reason: |
| --- | --- | --- | --- | --- |
| **1b** | Hunza | (01) Yes (02) No | No. times: | Reason: |
| **1c** | Bagrot | (01) Yes (02) No | No. times: | Reason: |
| **1d** | Astore | (01) Yes (02) No | No. times: | Reason: |
| **1e** | Rawalpindi/Islamabad | (01) Yes (02) No | No. times: | Reason: |
| **1f** | Karachi | (01) Yes (02) No | No. times: | Reason: |
| **1g** | Lahore | (01) Yes (02) No | No. times: | Reason: |
| **1h** | Peshawar | (01) Yes (02) No | No. times: | Reason: |
| **1i** | Other: | (01) Yes (02) No | No. times: | Reason: |
| **1j** | Outside Pakistan: | (01) Yes (02) No | No. times: | Reason: |

**2:** What language do you speak (i.e what is your mother tongue)? And what other languages?

- **Codes:** (01)Urdu, (**02**)Brusheski, (**03**)Shina, (04) English, (05) Other local languages (specify):, (06) Foreign languages (specify):
- **Note:** Multiple responses are possible.
- **Note:** Some confusion initially arose about this question. Respondents and the interviewers at first understood this question to ask what someone’s ‘mother tongue’ is, and therefore only marked one response. Later, this was corrected to include all languages one knows. Finally, in order to incorporate the benefits of knowing both one’s linguistic heritage and later life exposure, the mother tongue is underlined and starred, whereas other learned languages are simply circled.

**Section 8 - Other Activities:** This section determines the lifestyle of the people, the activities in which they are involved, various hobbies they have and the community programs and volunteer activates they perform.

**1:** What community activities are you involved in? (Note significant positions of leadership)

- **Codes:** (01) VO (Village Organization), (02) WO (Women’s Organization), (03) LSO (Local Support Organization), (04) SPO (Strengthening Participatory Organization), (05) Jamat Khana, (06) Madrasa, (07) Night School, (08) Fundraising for school or community, Other (specify):
- **Note:** If the individual is in a position of leadership within a community activity, note the activity and position.

**2:** What hobbies/interests do you have?

- **Codes:** (01) TV/movies (note language):, (02) Computer games or other digital games, (03) Internet, (04) Reading newspaper (note language):, (05) Reading books/stories (note language):, (06) Shopping, (07) Spending time with friends, (08) Tailor/silhay, (09) Cooking, (10) Athletics (note type and level of competition):, Other (specify):
- **Note on Codes:** Competitive athletic activity comes under (10), Amateur athletics will be (11)

**Note:** Ask what hobbies the individual regularly pursues, or what he/she does when he has free time.

**3:** What skills do you have (e.g. computer, electrical, artisanship, art, writing, athletics, etc.)?

- **Codes:** (01) Basic computer, (02) Programming, (specify):, (03) Graphic design, (04) Art (type):, (05) Electrical, (06) Mechanical, (07) Artisanship (e.g. carpentry, masonry, stitching; note type):, (08) Writing (note type and language):, Other (specify):
- **Note:** (01) Basic computer means Word, Excel, Internet, etc.

**4:** Would you be willing to have blood drawn for checking for anemia, high blood sugar or cholesterol?

- **Codes:** (01) Yes, (02) No
- **Note:** Explain that we will not be drawing blood for this study but may do so for a future study and would like to know if the participant would be willing to have blood drawn.

1. National Health and Nutrition Examination Survey (NHANES): Anthropometry Procedures Manual. Center for Disease Control. P 39. January 2007. http://www.cdc.gov/nchs/data/nhanes/nhanes_07_08/manual_an.pdf [↑](#footnote-ref-1)
2. National Health and Nutrition Examination Survey (NHANES): Anthropometry Procedures Manual. Center for Disease Control. P 46. January 2007. http://www.cdc.gov/nchs/data/nhanes/nhanes_07_08/manual_an.pdf [↑](#footnote-ref-2)
3. http://angrydr.blogspot.com/2006/06/laymans-guide-to-medical-equipment-3.html [↑](#footnote-ref-3)
