# Supplementary material for: Examining the relationships between early childhood experiences and adolescent and young adult health status in a resource-limited population: A cohort study
Source: PLoS Med. 2021 Sep 28;18(9):e1003745. doi: 10.1371/journal.pmed.1003745 (PMC8478204; doi:10.1371/journal.pmed.1003745)
Supplement: S3 Appendix — (PDF) [file pmed.1003745.s010.pdf]

 Participant ID: | | | | | | | | |

Form for Follow-Up of Young Adults from Oshikhandass نوجوانوں کا فلواپ کا فارم

Section No.1: بنیادی معلومات: (Basic Information)

1	Participant ID (New ID)	تحقیق میں حصہ لینے والے کا نیا شناختی نمبر	
2	Individual ID (Old ID)	تحقیق میں حصہ لینے والے کا پُرانا شناختی نمبر	
3	Date of Visit (DD/MMM/YY)	تاریخ ملاقات	________/________/________
4	Interviewer	انٹرویو لینے والے کا نام	
5	Name of Respondent (Official/Legal?)	تحقیق میں حصہ لینے والے کا نام (قانونی نام؟)	
5a	Recorded Name (Old)	نام پُرانا ریکارڈ کے مطابق	
5b	Nickname	نک نام	
8	Mother's Name	ماں کا نام	
9	Father's Name	باپ کا نام	
10	Reported DOB	تاریخ پیدائش رپورٹ کے مطابق	________/________/________
10a	Recorded DOB	تاریخ پیدائش رکارڈ کے مطابق	________/________/________
11	Age (from Reported DOB)	عمر (تاریخ پیدائش رپورٹ کے مطابق)	Years & Months ________ :مہینے ________ :سال
12	Address	موجودہ پتہ	
13	Contact Number	فون نمبر	
14	Email		
15	Raven's Matrices administered?	ریونز دیا گیا؟	(01)ہاں (02)نہیں<br>(01) Yes (02) No

Section No.2: جسمانی پیمائش: (Anthropometry)

1	Height (xxx. x cm)	قد(سنٹی میٹرز)	______ . __
2	Waist Girth (xxx .x cm)	کمر(سنٹی میٹرز)	______ .__
3	Weight (xxx.x kg)	وزن(کلوا گرام)	______ .__
4	Blood Pressure (mm Hg)	خون کا دباو(بلڈ پریشر)(ملی میٹر مرکری)	__ ____/______

Section No.3: صحت اور محل وقوع سے متعلق سوالات : (Health and Geography Questions)

1	آپ اپنی صحت کو کس طرح سے دیکھتے ہیں؟ آپ اپنی صحت کا تعین درج ذیل میں سے کس طرح کریں گے؟<br>How would you characterize your health in general?	(01)شاندار/بہت اچھی(02)اچھی(03)ٹھیک ٹھاک/مناسب(04)خراب(05)بہت خراب<br>(01) Excellent (02) Good (03) Satisfactory (04) Poor (05) Very poor

	اگر خراب / بہت خراب، تو معلوم کریں، کیوں؟<br>If poor/very poor, explore why:	1a
(01) شاندار / بہت اچھی (02) اچھی (03) ٹھیک ٹھاک / مناسب (04) خراب (05) بہت خراب<br>(01) Excellent (02) Good (03) Satisfactory (04) Poor (05) Very poor	آپ بچپن میں (پانچ سے پندرہ کی عمر تک) اپنی صحت کی حالت کا تعین کیسے کریں گے؟<br>How would you characterize your health as a child (ages 5-15)?	2
	اگر خراب / بہت خراب، تو معلوم کریں، کیوں؟<br>If poor/very poor, explore why:	2a
(01) دمہ (02) متواتر نمونیہ (03) کان کے بار بار انفیکشن (04) متواتر ہونے والے دست (05) آنتوں کے کیڑے (06) بلڈ پریشر سے متعلق مسائل (07) بڑا حادثہ (08) ہڈیوں کا ٹوٹنا (09) سرجری (10) دیگر (نشاندی کریں): ____________<br>(01) Asthma (02) Recurrent pneumonia (03) Repeated ear infections (04) Recurrent Diarrhea (05) Worms (06) Concerns about blood pressure: High/Low, (07) Major Accident (08)Broken bones (09) Surgery (10) Depression or other emotional problems (11) Other (specify): ____________	کیا آپ کو اپنی زندگی کے دوران کبھی صحت سے متعلق مخصوص قسم کے مسائل در پیش ہوئے ہیں؟<br>Have you had any specific health problems in your life?	3
(02) نہیں (01) ہاں<br>(01) Yes (02) No	کیا آپ ہسپتال میں داخل رہے ہیں؟<br>Have you ever been hospitalized?	4
	If yes (you have been hospitalized), where and for what? اگر ہاں تو کہاں اور کس لئے؟	4a

ہسپتال میں داخل ہونے کی تاریخ<br>Date of hospitalization	ہسپتال سے فارغ ہونے کی تاریخ<br>Date of discharge	ہسپتال کا نام<br>Name of hospital (place)	مسلہ کیا تھا؟ (ایک سے زیادہ جوابات اسکتے ہیں)<br>For what problem (more than one response possible)	نتیجہ<br>Outcome(s)<br>1 = علاج ہو گیا (cured), 2 = علاج کی کوشش کیا لیکن نہیں ہوا (treated but not cured), 3 = LAMA	
					A
					B
					C
					D

میڈیکل تاریخ کے بارے میں دیگر تاثرات، اگر ہسپتال میں داخل ہوئی ہیں یا نہیں:<br>Other comments about medical history, whether or not hospitalized:		4b
(02) نہیں (01) ہاں<br>(01) Yes (02) No	کیا آپ محسوس کرتے ہیں کہ آپ کو اشکنداس میں حفظان صحت / صحت کی دیکھ بال کی کافی و مناسب سہولیات تک رسائی حاصل ہے؟<br>Do you feel like you have access to adequate healthcare in Oshikhandass?	5
__________ km	اگر نہیں تو آپ یا آپ کے گھر کے افراد کو صحت کی سہولیات تک رسائی کے لئے کتنا (کلو میٹرز) سفر کرنا ہوتا ہے؟<br>If no, how far (in km) do you or your family travel for healthcare?	5a
(01) گلگت (02) اسلام آباد (03) لاہور (04) کراچی (05) دیگر (وضاحت کریں):<br>______________________________<br>(01) Gilgit (02) Islamabad (03) Lahore (04) Karachi (05) Other (specify): ______________________	صحت کی سہولیات تک رسائی کے لئے آپ کون سے شہر کا سفر کرتے ہیں؟<br>Which cities have you traveled to for healthcare?	5b
(01) ہاں (02) نہیں<br>(01) Yes (02) No	کیا آپ اب بھی اشکنداس میں رہتے ہیں؟<br>Do you still live in Oshikhandass?	6
**اگر سوال نمبر 6 کا جواب "ہاں" ہے، تو سیکشن 4 میں جائیں**<br>**If "Yes" to Question 6, skip to Section 4**		
	آپ کہاں رہتے ہیں؟<br>Where do you live now?	7
Years & Months __________ :مہینے __________ :سال	آپ اس نئے شہر میں کتنے عرصے سے رہ رہے / رہی ہیں؟<br>How long have you lived in your new city?	7a
	کس عمر میں آپ اشکنداس سے کسی دوسری جگہ منتقل ہوئے تھے؟<br>At what age did you initially move outside Oshikhandass?	8
(01) اسکول (02) روزگار / کام (03) شادی ہو گیا (04) خاندانی مسائل (05) دیگر (وضاحت کریں): ______________________<br>(01) School (02) Work (03) Married (04) Family Reasons (05) Other (specify): ________________	پہلے منتقل ہونے کے وجوہات / جگہ چھوڑنے کی وجہ؟ (ایک سے زیادہ جوابات آسکتے ہیں)<br>Reason(s) for initially moving (more than one response possible)	8a

(01)ہاں (02)نہیں<br>(01) Yes (02) No	کیا آپ محسوس کرتے ہیں کہ آپ کو اپنے شہر میں حفظان صحت / صحت کی دیکھ بھال کی کافی و مناسب سہولیات میسر ہے؟<br>Do you feel like you have access to adequate healthcare in your city?	9
	اگر نہیں تو آپ یا آپ کے گھر کے افراد کو صحت کی سہولیات تک رسائی کے لئے کتنا کلو میٹر (km) سفر کرنا ہوتا ہے؟<br>If no, how far (in km) do you or family travel for healthcare?	9a
**(General Questions) :عام سولات:Section No. 4**		
(01)ہاں (02)نہیں<br>(01) Yes (02) No	آپ شادی شدہ ہے؟<br>Are you married?	1
	اگر ہاں تو آپ کے شادی کئے ہوے کتنا عرصہ ہوا ہے؟<br>If yes, for how many years?	1b
**اگر شادی کی مدت نو ماہ سے کم ہے، تو سوال نمبر 3 پوچھیں**<br>**If not married 9 months or more, skip to Question 3**		
(01)ہاں (02)نہیں<br>(01) Yes (02) No	کیا آپ کے بچے ہیں؟<br>Do you have children?	2
	اگر ہاں تو آپ کے کتنے بچے ہیں؟ If yes, how many children do you have?	2a

عمر (سال اور مہینے میں)<br>Age (years and months)	جنس: (01) مرد (02) عورت<br>Gender: (01) Male (02) Female	سکول جا رہے ہیں؟ (01)ہاں (02) نہیں<br>Attending school? (01) Yes (02) No	کون سا سکول جا رہے ہیں:<br>If attending school, which school:	اگر نہیں جا رہے ہیں تو وجہ کیا ہے؟ (01) چھوٹے ہے (02) خرچہ زیادہ ہے (03) کوئی اور (بیان دیں)<br>If not attending reason(s)? (01) Too young (02) Could not afford (03) Other (specify)	
					A
					B
					C
					D

(01) کل وقتی طالب علم (02) جز وقتی طالب علم (03) کل وقتی طالب علم اور بر سر روز گار (04) جز وقتی طالب علم اور روز گار (05) تعلم مکمل ہوا ہے اور روز گار (06) تعلم مکمل ہوا ہے اور بے روز گار (07) گھر میں بغیر مزدوری کے کام کرتے ہیں<br>(01) Full-time student (02) Part-time student (03) Full-time student & employed (04) Part-	آپ کیا کرتے ہیں؟<br>What are you doing now?	3

time student & employed (05) Completed studies & employed (06) Completed studies & unemployed (07) Not in labor force & doing unpaid family work		
سال:__________ مہینے:__________<br>Years & Months	آپ کتنے عرصے میں اس مقام تک پہنچے؟<br>How long has this been your status?	3a
**اگر طالب علم ہے جو بر سر روزگار نہیں ہے (01-04)، تو سیکشن 6 میں جائیں؛ وارنہ، سیکشن 5 پوچھیں**<br>**If student who is not working (01-04), skip to Section 6; otherwise, administer Section 5**		
**Section No. 5: روزگار سے متعلق سوالات**<br>**Section No. 5 Employment questions**		
(01) بر سر روزگار ہے (02) پچھلے چھ مہینے کے دوران بر سر روزگار تھا لیکن اب نہیں ہے (03) پچھلے چھ مہینے کے دوران بر سر روزگار نہیں ہوں<br>(01) Currently employed (02) Employed within last 6 months, but not currently employed (03) Not employed in last 6 months	کیا آپ اس وقت بر سر روزگار ہے یا پچھلے چھ مہینوں کے دوران بر سر روزگار رہے ہیں؟<br>Are you currently employed or have you been employed in the last 6 months?	1
**اگر پچھلے چھ مہینے کے دوران بر سر روزگار نہیں تھا، سوال نمبر 3 پوچھیں: (If "Not employed in last 6 months" (03), skip to Question 3)**<br>**اگر ابھی بر سر روزگار ہے، سوال نمبر 5 پوچھیں: (If "Currently employed" (01), skip to Question 5)**		
(01) زبردستی نکالا گیا (02) دیوالیہ کمپنی کی مالی حالت کمزور ہونی کی وجہ سے ایک ہی بار بہت سے ملازموں کو نکالا گیا (03) روزگار سے خوش نہیں تھا (نوکری کو چھوڑنا اپنی مرضی تھی) (04) نوکری تبدیل کرنا چاہتا تھا (05) دیگر (وضاحت کریں):<br>__________________<br>(01) Fired (02) Laid off (03) Unhappy at job (left by choice) (04) Want to switch jobs (05) Other (specify):	اگر آپ پچھلے چھ مہینوں کے دوران بر سر روزگار رہے ہیں لیکن ابھی بر سر روزگار نہیں ہے تو آپ نے اپنی ملازمت کیوں چھوڑ دی؟<br>If you were employed in the last 6 months, but are not employed now, why did you end your job?	2
(01) ہاں (02) نہیں<br>(01) Yes (02) No	اگر آپ بر سر روزگار نہیں ہیں، تو کیا آپ کوئی کام کرنے کیلئے دستیاب ہیں؟<br>If not employed, are you available for work?	3
(01) ہاں (02) نہیں<br>(01) Yes (02) No	اگر آپ بے روزگار ہیں تو کیا آپ کوئی ملازمت کی تلاش کر رہے ہیں؟<br>If not employed, are you looking for work?	4
**اگر سوال نمبر 4 کا جواب "نہیں" ہے، سوال نمبر 12 میں جائیں**<br>**If "No" to Question 4, skip to Question 12**		
Years & Months سال:__________ مہینے:__________	اگر ہاں تو آپ کب سے ملازمت کی تلاش کر رہے ہیں؟<br>If yes (you are not employed and have been looking for work), how long have you been looking for?	4a
	آپ کا شعبہ عمل کیا ہے<br>What is/was your field of work?	5

	آپ کے کمپنی کا نام کیا ہے؟/ آپ کے ادارے کا نام کیا ہے؟<br>What is your business or organization's name?	6
(01) سروس/ادارہ (02) کاروباری (03) اپنا یا خاندان کا کاروباری<br>(04) دیگر (وضاحت کریں): ____________<br>(01) Service/organization (02) Business (03) Personal or family business (04) Other (specify):	یہ کس قسم کا ادارہ ہے؟<br>What type of organization is this?	6a
	آپ کا عہدہ کیا ہے؟<br>What is your job title?	7
	آپ کی ماہانہ آمدنی کیا ہے؟<br>What is your monthly income?	8
	آپ ایک ہفتے میں کتنے گھنٹے کام کرتے ہیں؟<br>How many hours per week are you working?	9
(01) ہاں (02) نہیں<br>(01) Yes (02) No	کیا آپ مزید کام کی تلاش کر رہے ہیں؟<br>Are you looking for more work?	10
(01) ہاں (02) نہیں<br>(01) Yes (02) No	کیا آپ اپنی موجودہ ملازمت کے عہدے سے مطمعن ہیں؟<br>Are you currently satisfied with your employment status?	11
(01) ہاں (02) نہیں<br>(01) Yes (02) No	کیا آپ کسی اور شعبہ روزگار سے وابستہ ہونے کی امید رکھتے ہیں؟<br>Do you hope to enter a different field of employment?	12
	اگر ہاں تو کون سا شعبہ میں؟<br>If yes, what field?	12a
(01) ہاں (02) نہیں<br>(01) Yes (02) No	کیا آپ کی آمدنی کے علاوہ کوئی دوسرا ذریعہ ہے خود کی مالی امداد کے لئے؟ (مثلاً ذاتی کارو بار، قرض، سرمایہ کاری، کرایہ سے آمدنی، وغیرہ)<br>کیا آپ کے پاس موجودہ ذریعہ کے علاوہ اپنی مالی معاونت کیلیے اور کوئی ذرائع ہیں؟ (مثلاً ذاتی کاروبار، قرض، سرمایہ کاری، کرایہ کا آمدنی، وغیرہ)<br>Do you have any other ways of supporting yourself financially other than in Question 1 above (e.g. other personal business, loan , investment, rental revenue)?	13
	اگر ہاں تو بیان کریں؟<br>If yes, describe:	13a
(01) ہاں (02) نہیں<br>(01) Yes (02) No	کیا آپ بغیر تنخواہ کے کوئی کام کرتے ہیں؟<br>Do you have any other unpaid work you do?	14
	اگر ہاں تو بیان کریں؟	14a

	If yes, describe:	
ہاں(01) نہیں(02)<br>(01) Yes (02) No	کیا آپ نے کبھی کوئی مالی قرض لیا ہے؟<br>Do you have any financial debt?	15
	اگر ہاں، تو کتنا؟<br>If yes, how much?	15a

تعلیم سے متعلق سوالات :Section No. 6

Section No.6: Education Questions

ہاں(01) نہیں(02)<br>(01) Yes (02) No	کیا آپ نے کبھی کوئی کلاس چھوڑا ہے؟<br>Did you ever skip any classes?	1
	اگر ہاں تو بیان کریں (مثلاً،2،3)؟<br>If yes, specify classes (e.g. 1st, 2nd, 3rd, etc.):	1a
ہاں(01) نہیں(02)<br>(01) Yes (02) No	کیا آپ نے کبھی کوئی کلاس دہرایا ہے؟<br>Did you ever have to repeat any classes?	2
	اگر ہاں تو بیان کریں؟ کہ کونسی کلاس کو کتنے مرتبہ دہرایا ہے؟<br>If yes, specify classes and number of times:	2a

کلاس جس کو دہرایا (مثلاً پریپ، 1، 2، وغیرہ)<br>Class Repeated (e.g. Prep, 1st, 2nd, etc.)	کتنے مرتبہ دہرایا (مثلاً، 2، 3، وغیرہ)<br>Number of Times Repeated (e.g. 1, 2, 3, etc.)	
		A
		B
		C
		D

ہاں(01) نہیں(02)<br>(01) Yes (02) No	کیا آپ کو کبھی سپلیمنٹری کا امتحانات دینے پڑے ہیں؟<br>Did you ever have to take any supplementary exams	3
	اگر ہاں تو بیان کریں؟ کہ کونسی کلاس اور کون سے کتابوں کی:<br>If yes, specify years and subjects:.	3a

امتحان کا سبجیکٹ<br>Subject of Exam (e.g. English, Physics, Islamiat, etc.)	کونسی کلاس میں سپلیمنٹری لی گئی<br>Year of Supplementary (e.g. Intermediate year 2, University year 1, etc.	کتنے مرتبہ لی گئی<br>Number of times taken	
			A
			B
			C

			D
			E

(02)نہیں (01)ہاں<br>(01) Yes (02) No	کیاآپ نے کبھی انگریزی کی کلاس لی ہے؟<br>Did you ever take an English class?	4
	اگرہاں توکونسی گریڈ/کلاس سے(مثلاًپریپ،1،2،3،وغیرہ)<br>If yes, from which grade level (e.g. Prep, 1, 2, 3, etc.)?	4a

5

برائےمہربانی وضاحت کریں کہ آپ نے پرائمری اسکول سے لیکریونیورسٹی یاپروفیشنل ٹریننگ،اعلی درجے کی تعلیم، جس میں ڈگریاں اور سرٹیفیکٹ کہاں سے حاصل کیاہے؟

Please specify where you attended school from Primary through University or Professional Training, up through your highest level of education, including degrees and certificates obtained:

مندرجہ ذیل کوڈ استعمال کریں اگلے تین صفحات پر ٹیبل کے لئے:

Use the following codes for the table on the next three pages:

1) ادارہ کانام لکھیں: مثلاً کراچی یونیورسٹی؛ آغاخان ڈائمنڈ جوبلی اسکول،اوش

Write the name of the institution e.g. Karachi University; Aga Khan Diamond Jubilee School, Oshikhandass

2) ادارہ کی قسم:مثلاًگورئمنٹ،SEKA،پرائیوٹ،مدرسہ،یادیگر(وضاحت کریں)

Institution Type: e.g. Government, AKES, private, madrasa, or other (specify)

3) شامل ہونے کے سال:مثلاً، 20102012؛ PI-2010 اگرابھی اسکول کی تعلیم چل رہاہے

Years Attended: e.g. 2010-2012; 2010-IP for schooling still in progress

4) ڈگری کی قسم:مثلاً،MlihPپریونیورسٹی؛cSBپررکالج؛cSFپرانٹرمیڈیٹ؛سرٹیفیکیٹ پر میٹرک،مڈل اسکول،یاپرائمری اسکول

Degree Type: e.g. MPhil for University; BSc for College; FSc for Intermediate; Certificate for Matric, Middle School, or Primary School

5) تعلیم کی شعبہ/سبجیکٹ:مثلاًکیمسٹری(بیچلرزیااس سے اوپر کے لئے)؛سائنس یاآرٹس(میٹرک اورانٹرمیڈیٹ کے لئے)

Area of Study/Subject: e.g. Chemistry (for Bachelor's or above); Science or Arts (for Matric and Intermediate)

6) بورڈکانام:مثلاًUIK،فیڈرال اسلام آباد،کراچی،پنجاب،آغاخان،یادیگر(وضاحت کریں)

Board: e.g. KIU, Federal – Islamabad, Karachi, Punjab, Aga Khan, or other (specify)

7) آخری گریڈ:مثل ًمثلاً"A"یا"B"؛اگرمعلوم ہے،فیصد لکھیں اردو،انگریزی،ریاضی،اورڈگری کی شعبہ کے لئے

Final Grade: e.g. A or B; record percentages for degree subject, Urdu, English, and Math if known

8) پچھلااسکول سے تبدیل کرنے کی وجہ(اگرکوئی تبدیلے ہوئی):(01)بہتراسکول(02)زیادہ قابل استطاعت (03)گھرسے زیادہ قریب(04)خاندان منتقل کردیا(05)دیگر (وضاحت کریں)

Reason for Change From Previous School (if any change): (01) Better school (02) More affordable (03) Closer to home (04) Family moved (05) Other (specify)

	1	2	3	4	5	6	7	8
ادارہ کا سطح<br>Institution Level	ادارہ کا نام<br>Institution Name	ادارہ کا قسم<br>Institution Type	شامل ہونے کے سال<br>Years Attended	ڈگری کی قسم<br>Degree Type	شعبہ تعلیم<br>Area of Study	بوڈر کا نام<br>Board	گریڈ<br>Final Grade	اسکول تبدیلی کی وجہ<br>School Change
پیشہ ورانہ تربیت اور دیگر سرٹیفیکیٹ/ڈپلوما<br>Professional Training and Other Certificates / Diplomas (LHW/PTC/CT/Montessori/madrasa/etc)								
ماسٹرز یا اس سے اوپر کے لئے یونیورسٹی<br>University for Master's or above (MA/MSc/MBA/MPhil/PhD/etc)								
بیچلرز کے لئے کالج یا یونیورسٹی<br>College or University for Bachelor's (BA/BSc/BComm/etc)								

	1	2	3	4	5	6	7	8
ادارہ کا سطح<br>Institution Level	ادارہ کا نام<br>Institution Name	ادارہ کا قسم<br>Institution Type	سال میں شرکت<br>Years Attended	ڈگری کی قسم<br>Degree Type	شعبہ تعلیم<br>Area of Study	بوڑد کا نام<br>Board	گریڈ<br>Final Grade	اسکول تبدیلی کی وجہ<br>School Change
انٹرمیڈیٹ<br>Intermediate (FA/FSc/IComm)								
میٹرک رہائی اسکول(9-10)<br>Matric/High School (9-10)							Overall:<br>English:<br>Math:<br>Urdu:<br>Other (specify):	
مڈل اسکول(6-8)<br>Middle School (6-8)								

	1	2	3	4	5	6	7	8
ادارہ کا سطح<br>Institution Level	ادارہ کا نام<br>Institution Name	ادارہ کا قسم<br>Institution Type	سال میں شرکت<br>Years Attended	ڈگری کی قسم<br>Degree Type	شعبہ تعلیم<br>Area of Study	بوڈر کا نام<br>Board	گریڈ<br>Final Grade	اسکول تبدیلی کی وجہ<br>School Change
پرائمری اسکول (پریپ-5)<br>Primary (prep-5)								
کیا مکمل کرلیا گیا تعلیم کے اعلی درجے کے لئے سرٹیفکیٹ یا ڈگری مشاہدہ کیا ہے؟<br>Has the certificate or degree for the highest level of education completed been observed?	(01) ہاں (02) نہیں (03) ابھی پڑھائی چل رہی ہے<br>(01) Yes (02) No (03) In Progress (IP)							

تاثرات (مثلاً اگر تعلیم کے دوران اسکول جانا روک دیا، انعام یا اعزاز حاصل کیے گے، یا دوسرے مشکلات):

Comments (e.g. if break taken in education, awards or honors received, or other hardships, please take note):

(00) نرسری/پریپ (98) کبھی نہیں داخل ہوا (99) تعلیم چل رہی ہے؛ ورنہ، کلاس/گریڈ کی سطح لکھیں: ---------------<br>(00) Nursery/prep (98) Never enrolled (99) Ongoing education; Otherwise, write grade level:	آپ نے کس سطح پر تعلیم کو روک دیا؟<br>At what class/grade level did you stop your education?	6
**If "Ongoing education" (99), skip to Question 8**		
(98) کبھی نہیں داخل ہوا (99) تعلیم چل رہی ہے؛ ورنہ، عمر لکھیں<br>(98) Never enrolled (99) Ongoing education; Otherwise, write age:	آپ نے کس عمر میں اپنی تعلیم کو روک دیا تھا؟<br>At what age did you stop your education?	7
(01) دلچسپی نہیں تھا (02) مالی کا متحمل نہیں ہو سکتا (03) خاندان کے لئے مالی طور پر کمانے کی ضرورت پڑی (04) جسمانی معذوری (05) بیماری (06) دیگر (وضاحت کریں):<br>______________________<br>(01) Was not interested (02) Could not afford (03) Needed to earn for family (04) Physical disability (05) Illness (06) Other (specify):	ایک دفعہ آپ نے تعلیم کو روک دیا تو وہ کونسے وجوہات تھے جس کی وجہ آپ کو اپنی تعلیم کو روکنا پڑا؟ (اخراجات برداشت نہیں کر سکتے تھے، دلچسپی نہیں تھی، جسمانی معذوری کی وجہ سے، بیماری کی وجہ سے یا کوئی اور وجہ سے؟)<br>Once you stopped your education, why did you not pursue further education?	7a
(01) پرائمری (Primary) (02) مڈل (Middle) (03) میٹرک/ہائی اسکول (High school/Matric) (04) FA/FSc/FComm (05) BA/BSc/BComm (06) MA/MSc/MBA (07) MPhil/PhD (08) پیشہ ورانہ تربیت اور دیگر سرٹیفیکیٹ/ڈپلوما (Professional training) (LHW/PTC/CT/Montessori/madrasa/etc)	ابھی تک کس سطح پر تعلیم حاصل کر چکے ہیں یا ابھی کر رہے ہیں؟<br>Which education level has been completed or is in progress?	8
(01) پرائمری (Primary) (02) مڈل (Middle) (03) میٹرک/ہائی اسکول (High school/Matric) (04) FA/FSc/FComm (05) BA/BSc/BComm (06) MA/MSc/MBA (07) MPhil/PhD (08) پیشہ ورانہ تربیت اور دیگر سرٹیفیکیٹ/ڈپلوما (Professional training) (LHW/PTC/CT/Montessori/madrasa/etc)	اگر آپ کی تعلیم چل رہی ہے، کس سطح تک آپ کا تعلیم حاصل کرنے کا ارادہ ہے؟<br>If ongoing education, which level of education do you intend to study toward?	9

(Travel History) سفر کے بارے میں معلومات :Section No. 7

			کیا آپ نے کبھی ان شہروں/علاقے میں سفر کیا ہے؟<br>Have you been to any of these cities/areas?	1
(01) گلگت<br>(01) Gilgit	(01) ہاں (02) نہیں<br>(01) Yes (02) No	ایک مہینہ میں کتنی مرتبہ:<br>No. times per month	وجہ:<br>Reason:	1a
(02) ہنزہ<br>(02) Hunza	(01) ہاں (02) نہیں<br>(01) Yes (02) No	کتنی مرتبہ:<br>No. times:	وجہ:<br>Reason:	1b
(03) بگروٹ<br>(03) Bagrot	(01) ہاں (02) نہیں<br>(01) Yes (02) No	کتنی مرتبہ<br>No. times:	وجہ:<br>Reason:	1c
(04) استور<br>(04) Astore	(01) ہاں (02) نہیں<br>(01) Yes (02) No	کتنی مرتبہ:<br>No. times:	وجہ:<br>Reason:	1d

(05)روالپنڈی/اسلام آباد<br>(05) Rawalpindi/Islamabad	(01)ہاں (02)نہیں<br>(01) Yes (02) No	کتنی مرتبہ:<br>No. times:	وجہ:<br>Reason:	1e
(06)کراچی<br>(06) Karachi	(01)ہاں (02)نہیں<br>(01) Yes (02) No	کتنی مرتبہ:<br>No. times:	وجہ:<br>Reason:	1f
(07)لاہور<br>(07) Lahore	(01)ہاں (02)نہیں<br>(01) Yes (02) No	کتنی مرتبہ:<br>No. times:	وجہ:<br>Reason:	1g
(08)پشاور<br>(08) Peshawar	(01)ہاں (02)نہیں<br>(01) Yes (02) No	کتنی مرتبہ:<br>No. times:	وجہ:<br>Reason:	1h
(09)کوئی دیگر بڑے شہر (بیان کریں):<br>(09) Other:	(01)ہاں (02)نہیں<br>(01) Yes (02) No	کتنی مرتبہ:<br>No. times:	وجہ:<br>Reason:	1i
(10)پاکستان سے باہر (بیان کریں):<br>(10) Outside Pakistan:	(01)ہاں (02)نہیں<br>(01) Yes (02) No	کتنی مرتبہ:<br>No. times:	وجہ:<br>Reason:	1j

(01)اردو (02)بروشسکی (03)شینا (04) انگریزی<br>(05)کوئی اور مقامی زبان (وضاحت کریں): ____________<br>(06)بیرونی زبانیں (وضاحت کریں): ____________<br>(01) Urdu (02)Brusheski (03)Shina (04) English<br>(05) Other local languages (specify): ____________<br>(06) Foreign languages (specify): ____________	آپ کون سی زبانیں بولتے ہیں؟<br>What languages do you speak?	2

Section No. 8: دوسری سرگرمیاں: (Other Activities)

(01) OV گاؤں تنظیم (02) OW خواتین کی تنظیم (03) OSL مقامی حمایت تنظیم (04) OPS شرکت کو مضبوط بنانے کی تنظیم (05) جماعت خانہ (06) مدرسہ (07) رات اسکول (08) اسکول یا کمیونٹی/برادری کے لئے فنڈ ریزنگ (09) دیگر (وضاحت کریں): ____________<br>(01) VO (Village Organization) (02) WO (Women's Organization) (03) LSO (Local Support Organization) (04) SPO (Strengthening Participatory Organization) (05) Jamat Khana (06) Madrasa (07) Night School (08) Fundraising for school or community (09) Other (specify):	آپ کن سماجی (community) سرگرمیوں سے وابستہ ہیں؟ (جماعت خانے، نائٹ اسکول، کھیل کود کے سرگرمیاں، چندہ اکٹھا کرنا)<br>What community activities are you involved in? (Note significant positions of leadership)	1
(01) VT/فلم (زبان نوٹ کریں): ____________ (02) کمپیوٹر پر کھیلنا یا دیگر ڈیجیٹل میڈیا (03) انٹرنیٹ (04) اخبار پڑھنا (زبان نوٹ کریں):<br>(05) کتابیں/کہانیاں پڑھنا (زبان نوٹ کریں): ____________<br>____________<br>(06) شاپنگ (07) دوستوں کے ساتھ وقت گزارنا (08) سلائی (09) کھانا پکانا (10) ایتھلیٹکس (قسم اور مسابقت کی سطح نوٹ کریں): ____________	آپ کے پسندیدہ مشاغل کیا ہیں؟<br>What hobbies/interests do you have?	2

(11)دیگر (وضاحت کریں): ____________________<br>(01) TV/movies (note language): _________________ (02) Computer games or other digital games (03) Internet (04) Reading newspaper (note language): ___________ (05) Reading books/stories (note language): __________ (06) Shopping (07) Spending time with friends (08) Tailor/silhay (09) Cooking (10) Athletics (note type and level of competition):_________________<br>(11) Other (specify): ___________________________		
(01) بنیادی کمپیوٹر (02) پروگرامنگ (وضاحت کریں): ______________ (03) گرافک ڈیزائن (04) آرٹ (قسم نوٹ کریں): ______________ (05) الیکٹریکل (06) میکانی (07) ہنر کارکن (مثل گارپینٹری، چنائی، سلائی؛ قسم نوٹ کریں): ______________ (08) لکھنا (قسم اور زبان نوٹ کریں): ______________<br>(09) دیگر (وضاحت کریں): ____________________________________<br>(01) Basic computer (02) Programming (specify): ________________ (03) Graphic design (04) Art (type): ________________ (05) Electrical (06) Mechanical (07) Artisanship (e.g. carpentry, masonry, stitching; note type): _________________________ (08) Writing (note type and language):___________________________________<br>(09) Other (specify):_____________________________________	آپ کے پاس کونسی فنی صلاحت موجود ہے؟<br>What skills do you have (e.g. computer, electrical, artisanship, art, writing, athletics, etc.)?	3
(02) نہیں (01) ہاں<br>(01) Yes (02) No	کیا آپ خون کی کمی، کولیسٹرول اور ہائی بلڈ شوگر کی جانچ پڑتال کیلیے خون دینے کیلیے راضی ہونگے؟<br>Would you be willing to have blood drawn for checking for anemia, high blood sugar or cholesterol?	4
