# Supplementary material for: Examining the relationships between early childhood experiences and adolescent and young adult health status in a resource-limited population: A cohort study
Source: PLoS Med. 2021 Sep 28;18(9):e1003745. doi: 10.1371/journal.pmed.1003745 (PMC8478204; doi:10.1371/journal.pmed.1003745)
Supplement: S4 Appendix — (DOCX) [file pmed.1003745.s011.docx]

**S4 Appendix Description of the psychometric analysis of the Raven’s Standard Progressive Matrices and Colored Progressive Matrices**

The Raven’s Combined Matrices measure (including Standard and Colored Matrices) was comprised of six sets (specified as A, AB, B, C, D, and E) of 12 visual matrices for a total of 72 multiple-choice items. Participants viewed the stimulus pattern and selected from one of six to eight options to correctly complete the pattern. Administration was done in an adaptive manner, meaning all participants began with item C1 and completed earlier items from sets A through B only if they demonstrated substantial difficulty with the more advanced set(s). See S8 Appendix Protocol on Raven’s Matrices Administration for further details.

The data were comprised of 1405 cases randomly divided into two subsamples for exploratory factor analyses (EFA; *n* = 700) and confirmatory factor analyses (CFA; *n* = 705). Scoring rules were applied as follows: if RCOMSA01 was answered correctly, Rav1 = 1, else Rav1 = 0; if an item was below starting point and not administered (e.g., RCOMSA01 = 999), full credit was awarded (Rav1 = 1); whereas for subsequent non-administered items above the discontinuation point (e.g., RSMSE01 = 999), no credit was awarded (Rav61 = 0).

The coefficient alpha reliability estimate for the full 72-item scale was sufficient at 0.77. Descriptive statistics and item characteristic curves were reviewed. Data were inspected for multicollinearity; items 1 through 26 were found to have variance inflation factors > 10 and tolerance < .20 which is indicative of substantial multicollinearity and consequently these items were necessarily dropped from analyses. The remaining 46 items were subjected to EFA.

Parallel analyses indicated up to 5 factors and scree inspection indicated up to 4 factors could plausibly be extracted for the 46 items submitted for analysis. The Kaiser-Meyer-Olkin measure of sampling adequacy was sufficient at 0.912, and Bartlett’s test of sphericity was significant, *X^2^* = 10,618.128, *df* = 1,035, *p* < 0.001. Principal components analyses were applied and one through four factors were considered. Thirty-one items loaded at the 0.32 level or higher on the single-factor solution selected.

Structural equation modelling was applied to test the reserved subsample to determine how well the single latent construct model fit the data. The unitary factor model fit the data satisfactorily, *X^2^* = 2,269.573 *df* = 434 *p* < 0.001, RMSEA = 0.077, and CFI = 0.968, further supporting the validity of the resultant Total Score. The reliability estimate for the revised Total Score comprised of the 31 items that loaded on the unitary factor the reliability estimate was excellent at 0.91. However, to preserve comparability to previously published research that utilized the summed total raw score for all 72 items of the Raven, this was selected for use in the current study. For interpretability of scores, raw scores were linearly transformed into T-scores (i.e., mean of 50 and a standard deviation of 10) based on sample distribution characteristics.
