# Supplementary material for: Examining the relationships between early childhood experiences and adolescent and young adult health status in a resource-limited population: A cohort study
Source: PLoS Med. 2021 Sep 28;18(9):e1003745. doi: 10.1371/journal.pmed.1003745 (PMC8478204; doi:10.1371/journal.pmed.1003745)
Supplement: S5 Appendix — (DOCX) [file pmed.1003745.s012.docx]

**S5 Appendix Protocol on Raven’s Standard Progressive Matrices and Colored Progressive Matrices Administration (Raven’s Combined Progressive Matrices, RCM)**

**Protocol based on MAL-ED Study**

MAL-ED Etiology, Risk Factors and Interactions of Enteric Infections and Malnutrition and the Consequences for Child Health and Development. Birth Cohort Studies: Manual of Procedures. [Unpublished]. Bethesda, MD: MAL-ED, November 2014.

# Purpose

The Raven’s Combined Progressive Matrices will be administered to each adolescent/young adult (in future referred to as adolescent) in order to assess nonverbal reasoning ability. We are collecting this information because it is one of our primary outcomes.

# Materials

Raven’s Combined Progressive Matrices form (RCM), RCM SOP, Raven’s Standard and Colored Progressive Matrices test books, clipboard, pen.

# Methods

In order to use a standardized administration, we have decided to use a combination of the Standard Progressive Matrices (SPM) and Colored Progressive Matrices (CPM). The SPM has 5 sets of problems (A – E) with each set containing 12 problems (1 – 12). The CPM has 3 sets of problems (A, AB, B) with each set containing 12 problems (1 – 12). We will start with set C of the SPM and if a particular adolescent does not answer the problems from start point to C5 correctly, the tester will go back 1 step to set B of the CPM. The rule to “go back 1 step” will continue as such (if the adolescent does not answer the problems from start point to B5 from the CPM correctly, s/he will be given set AB from the CPM, etc). So, sets A and B from the SPM will never be administered. An adolescent will receive credit for problems that came before his/her starting point (so, if s/he successfully completes the problems from start point to C5 from SPM, s/he will receive credit for all of the sets in the CPM.) The Research Coordinator (RC) administering the test should leave the record form blank for problems that the adolescent did not need to complete (the database management team will write a program that will automatically give the mothers credit for any problems that came before his/her starting point).

The Raven’s Combined Progressive Matrices will be administered to each mother by a Research Coordinator (RC). This will be done at the same time as the adolescent questionnaire is administered. In rare situations it may be allowable to do the Raven’s CPM at a different time, in which case, the date of test administration will be noted. It requires a quiet space with good lighting. Therefore, this test will be administered at the Research Study Office in Oshikhandass, or at the participant’s location if outside Oshikhandass. The RC and the participant will sit together in a quiet location. All efforts will be made to avoid any distractions. The RC will place the test booklet flat on the table, in front of the participant. The RC will introduce the Raven’s Combined Progressive Matrices using the following script. The items marked *say* are things the RC will say, those marked *do* are things the RC will do. All RC must use this exact script to ensure that every participant receives the same instructions.

say: This is a test of observation and clear thinking.

do: Show subject problem C1 (from the Standard Progressive Matrices).

say: The top part of C1 is a pattern with a bit cut out of it. Look at the pattern and think what the piece needed to complete the pattern correctly both along and down must look like. Then, find the right piece out of the 8 bits shown below. Only one of these pieces is perfectly correct. These are all…

do: Point to each in turn.

say: …the correct shape to fill the space but only one of them is the right pattern. Number 2 is not the right pattern; #4 is quite wrong; #1 is nearly right but is wrong here.

do: Point to the piece which is correct.

say: #8 is the right piece, isn’t it? So the answer is #8.

do: Write the correct answer on the answer sheet. do: Flip the page to problem C2.

say: Please point to the correct piece for this problem.

do: Allow sufficient time for subject to respond.

Make sure subject has answered correctly so you are confident s/he understands the instructions. Write the correct answer on the answer sheet. *If s/he responds incorrectly, go back to problem C1 and explain the instructions again. Please remember that after a second explanation of problem C1, the participant must go on and complete the rest of the test. That is, only 2 examples of how to solve problem C1 will be given to any 1 subject. Giving further instruction will invalidate the participant’s scores.*

say: On every page of the booklet there is a pattern with a piece missing. You have to choose the correct piece to complete each pattern. When you think you’ve found the right one, point to it. If you want to change your mind, let me know. Do you have any questions? You may work at your own pace. If you are not sure, guess. If you get stuck, let me know and we’ll move on to the next problems and then come back to those you had difficulty with. We will now begin.

do: Turn over to problem C3 and start (note the time in the ‘start time’ boxes on the record form). For *every problem in set C (C1 – C12), before the person taking the test has time to point to one of the pieces:*

say: Look carefully at these pieces (move your finger across them). Only one of these pieces is right to complete the pattern. Be careful. Look at the pattern and each of the 8 pieces first (point to each of the 8 pieces). Now you point to the right one to complete the pattern.

do: Write down the number of the piece that the participant points to as the answer. If they do not correctly answer from start point through C5 (they will all get problem C1 correct because you actually do it for them as part of the instructions), go back and administer set B from the Colored Progressive Matrices. If they do not correctly answer from start point to B5 (from the Colored Progressive Matrices), go back and administer set AB from the Colored Progressive Matrices. If they do not get from start point through AB5 (from the Colored Progressive Matrices) correct, go back and administer set A from the Colored Progressive Matrices.

Therefore, the order of test administration is as follows:

- Begin with set C of the Standard Progressive Matrices. If subject responds to problems from start point to C5 correctly, continue on to finish set C and then go on to sets D and E (she will receive credit for all of the problems that came before set C that she did not need to complete). If subject does not respond to problems from start point to C5 correctly:
- go back and administer set B from the Colored Progressive Matrices. If subject responds to problems from start point to B5 correctly, continue on to finish set B (from Colored Progressive Matrices) and then go on to sets C through E of the Standard Progressive Matrices (subject will receive credit for any problems that came before set B that she did not need to complete). If subject does not respond to problems from start point to B5 (from Colored Progressive Matrices) correctly:
- go back and administer set AB from the Colored Progressive Matrices. If subject responds to problems from start point to AB5 correctly, continue on to finish set AB (from Colored Progressive Matrices) and then go on to set B of the Colored Progressive Matrices and then sets C through E of the Standard Progressive Matrices (subject will receive credit for any problems that came before set AB that she did not need to complete). If subject does not respond to problems from start point to AB5 (from Colored Progressive Matrices) correctly:
- go back and administer set A from the Colored Progressive Matrices. Administer all problems in set A (from Colored Progressive Matrices) and then go on to sets AB and B of the Colored Progressive Matrices and then sets C through E of the Standard Progressive Matrices.

For all of the problems, if the participant is pointing to a choice (whether right or wrong) without taking his/her time to look at the pattern and each of the possible choices, remind his/her to take her time and to consider every choice before answering. At the end of 20 minutes, note the number of the problem being worked on but continue until the test is completed.

**Reporting**

Write the adolescent’s Participant ID in the space provided at the upper left corner.

When the RC starts the test s/he will start the stopwatch. S/he will note what number item the participant is solving after 20 minutes have passed. If the participant has already finished solving all the problems before 20 minutes the RC will also note this.

# Start Time

The RC will record the time the participant begins testing (testing begins when the participant starts problem C3) on a clock. The hour and minutes will be recorded separately using two digits.

**End Time**

The RC will record the time when the participant finished the last question on a . The hour and minutes will be recorded separately using two digits.

# Answers section A

For each item, the participant will indicate his/her answer choice to the RC by pointing. The RC will record the answers to each question on the record sheet in the box labeled with that question’s number. The answer to question A1 will be recorded in box A1, the answer to question A2 will be recorded in box A2. The participant will only solve the problems in set A if s/he is not able to correctly respond to problems from start point to AB5 (from the Colored Progressive Matrices). If s/he does not need to solve the problems in set A, write NA in the first box (A1) and strike through the remaining boxes in the section.

# Answers section AB

For each item, the participant will indicate his/her answer choice to the RC by pointing. The RC will record the answers to each question on the record sheet in the box labeled with that question’s number. The answer to question AB1 will be recorded in box AB1, the answer to question AB2 will be recorded in box AB2. The participant will only solve the problems in set AB if s/he is not able to correctly respond to problems from start point to B5 (from the Colored Progressive Matrices). If s/he does not need to solve the problems in set AB, write NA in the first box (AB1) and strike through the remaining boxes in the section.

# Answers section B

For each item, the participant will indicate his/her answer choice to the RC by pointing. The RC will record the answers to each question on the record sheet in the box labeled with that question’s number. The answer to question B1 will be recorded in box B1, the answer to question B2 will be recorded in box B2. The participant will only solve the problems in set B if /she is not able to correctly respond to problems from start point to C5 (from the Standard Progressive Matrices). If s/he does not need to solve the problems in set B, write NA in the first box (B1) and strike through the remaining boxes in the section.

# Answers section C

For each item, the participant will indicate her answer choice to the RC by pointing. The RC will record the answers to each question on the record sheet in the box labeled with that question’s number. The answer to question C1 will be recorded in box C1, the answer to question C2 will be recorded in box C2. The participant will solve all the problems in set C before proceeding to set D.

# Answers section D

For each item, the participant will indicate his/her answer choice to the RC by pointing. The RC will record the answers to each question on the record sheet in the box labeled with that question’s number. The answer to question D1 will be recorded in box D1, the answer to question D2 will be recorded in box D2. The participant will solve all the problems in set D before proceeding to set E.

**Answers section E**

For each item, the participant will indicate his/her answer choice to the RC by pointing. The RC will record the answers to each question on the record sheet in the box labeled with that question’s number. The answer to question E1 will be recorded in box E1, the answer to question E2 will be recorded in box E2. The participant will solve all problems in set E.

# Record the Section and Item number of the problem being worked on at end of 20 minutes

The RC will record the problem number the participant is solving when the 20th minute of her test ends. S/he will record the problem number using the codes xA01--xE12. If the participant is finished the test before 20 minutes are up the RC will record 9999.

**Raven’s Combined Matrices Scoring Form**

| **Raven’s Combined Matrices (RCM)** | |
| --- | --- |
| Participant Name and ID |  |
| Research Coordinator ID |  |
| Date (DD/MMM/YY) |  |
| Start Time (HH:MM) |  |
| End Time (HH:MM) |  |
| Question being worked on at 20 min (if value is less than 4 characters, precede it with an x, e.g. problem C5 would be xC05; if entire test is completed before 20 minutes, record 9999) |  |
| **Raw Score Total:** |  |

| **Raven’s Colored Matrices (range of responses is 1-6)** | | | |
| --- | --- | --- | --- |
| **#** | **Section A** | **Section AB** | **Section B** |
| 1 |  |  |  |
| 2 |  |  |  |
| 3 |  |  |  |
| 4 |  |  |  |
| 5 |  |  |  |
| 6 |  |  |  |
| 7 |  |  |  |
| 8 |  |  |  |
| 9 |  |  |  |
| 10 |  |  |  |
| 11 |  |  |  |
| 12 |  |  |  |
| **Section Total:** |  |  |  |
| **Raven’s Standard Matrices (range of responses is 1-8)** | | | |
| **#** | **Section C** | **Section D** | **Section E** |
| 1 |  |  |  |
| 2 |  |  |  |
| 3 |  |  |  |
| 4 |  |  |  |
| 5 |  |  |  |
| 6 |  |  |  |
| 7 |  |  |  |
| 8 |  |  |  |
| 9 |  |  |  |
| 10 |  |  |  |
| 11 |  |  |  |
| 12 |  |  |  |
| **Section Total:** |  |  |  |
