# Supplementary material for: Examining the relationships between early childhood experiences and adolescent and young adult health status in a resource-limited population: A cohort study
Source: PLoS Med. 2021 Sep 28;18(9):e1003745. doi: 10.1371/journal.pmed.1003745 (PMC8478204; doi:10.1371/journal.pmed.1003745)
Supplement: Attachment — Submitted filename: rasmussen.pdf [file pmed.1003745.s013.pdf]

This paper describes a long–term follow–up of children in rural Pakistan.

I was asked for a statistical report and I interpret that to include all aspects of the design and conduct of the study.

Points of detail

Page 8 The concept of a T score is not one widely used in health and I have seen it lead to confusion so perhaps add that it means standardised to mean 50 and standard deviation 10.

Page 8 In fact Figure 3 seems to suggest that BMI was used as a continuous variable (which is the superior way to incorporate it) so perhaps this needs clarifying here.

Page 8 As written here anyone with systolic ≤ 120 would not be classified. Table 1 suggests otherwise.

Page 9 If the score on the Raven's is a count of positive responses as page 8 states then why re–scale it and then square it when a log linear model could be used? That would be the usual way of constructing a model for counts.

Page 10 I am afraid I did not grasp the sentence starting 'Of 126'. Figure 1 is clearer in this respect.

Page 10 If the study participants have a mean age of 22.6 why use 19 in computing HAZ? I can guess but perhaps clarify.

Page 10 If you have adolescent HAZ why correlate childhood HAZ with adolescent height not HAZ?

Page 11 Does this mean that adjusting for age and sex English speakers had had 2.4 years more education? As it is worded it looks as though it is saying people with equivalent actual years of education act as though they had 2.4 years more if they also spoke English. At least that is how I initially read it.

Page 12 As a very minor point I would write t–test in lower case to avoid any possible confusion with T–score. I assume Hotelling's T^2 is not meant.

Page 12 If the 49 people who do not speak Shina are unusual in some way I wonder whether any apparent relationship with that variable is driven by possible outlying values?

Page 13, caption to Figure 2 The usual formula for BIC involved penalising minus twice the log likelihood by adding $k \ln n$ so I am not sure what log BIC would mean.

Figure 3 Is the y–axis the squared T–score or the raw score, or the T–score?

Supplement S1 This reveals that what was used as the score was not the score on the original full scale. Without more information on the items which were dropped it is hard to assess how this affects the conclusions. Some of the appendix is going to be incomprehensible to people who are not familiar with administration of tests. I assume it is run in an adaptive way and if you fail a sequence of harder items you are auto–scored zero above that and vice versa for easy items but perhaps that can be clarified.

Supplement Table 4 and 5 I can see why some cells here are empty but do the others represent some form of variable subset selection? If so this needs reporting in the methods. Selecting a subset of variables in a way driven by the data leads to a model which is unlikely to replicate (Babyak, 2004). Screening variables to enter the model has been criticised too (Sun et al., 1996)

Incorporating height and weight and BMI in the model seems rather strange given the artefactual relationship between them. The authors have excluded other variables from the network on theoretical grounds so I wonder why a theory–based choice was not made here.

Point of more substance

The authors remark that there had been many positive social changes in the village in the intervening years and I note that the distribution of HAZ is much improved between time points. Excellent thing though this undoubtedly is for the children is it another limitation to interpretation of the childhood predictors as they have been swamped by everyone being exposed to a more supportive environment? This would not alter the value of the study but it is perhaps worth mentioning.

Summary

Mostly points for clarification.

Michael Dewey

References

M A Babyak. What you see may not be what you get: a brief, nontechnical introduction to overfitting in regression–type models. *Psychosomatic Medicine*, 66:411–421, 2004.

G-W Sun, T L Shook, and G L Kay. Inappropriate use of bivariable analysis to screen risk factors for use in multivariable analysis. *Journal of Clinical Epidemiology*, 49:907–916, 1996.
