# Supplementary material for: Examining the relationships between early childhood experiences and adolescent and young adult health status in a resource-limited population: A cohort study
Source: PLoS Med. 2021 Sep 28;18(9):e1003745. doi: 10.1371/journal.pmed.1003745 (PMC8478204; doi:10.1371/journal.pmed.1003745)
Supplement: Attachment — Submitted filename: Responses_to_Reviewers_Final.docx [file pmed.1003745.s014.docx]

| **Reviewer** | **Comment** | **Response** | **Change with Line numbers (L) in Marked Up Version** |
| --- | --- | --- | --- |
| **Editor** | Please revise your title according to PLOS Medicine's style. Your title must be nondeclarative and not a question. It should begin with main concept if possible. Please place the study design (e.g. "A prospective cohort study,") in the subtitle (i.e., after a colon) | We have changed the title | Title |
|  | Abstract summary - At this stage, we ask that you reformat your non-technical Author Summary. The Author Summary should immediately follow the Abstract in your revised manuscript. This text is subject to editorial change and should be distinct from the scientific abstract. The summary should be accessible to a wide audience that includes both scientists and non-scientists. Please see our author guidelines for more information. https://journals.plos.org/plosmedicine/s/revising-your-manuscript#loc-author-summary | We have added the author summary | L 91-125 |
|  | **In the abstract Methods and Findings** |  |  |
|  | Please ensure that all numbers presented in the abstract are present and identical to numbers presented in the main manuscript text. | We have reviewed and revised numbers | Abstract  L 64-77 |
|  | Please quantify the main results with both 95% CIs and p values. | Following discussion with the editors, we have revised the reporting in the abstract and main text | Abstract  L 68-76 |
|  | Please include the important dependent variables that are adjusted for in the analyses. | Our primary analysis was a Bayesian Network. As a method for structure discovery, this includes all (important) variables, which can be potentially independent and dependent variables. Following discussion with the editors, we have reviewed the text for clarity | No changes |
|  | In the last sentence of the Abstract Methods and Findings section, please describe the main limitation(s) of the study's methodology. | We have added to the abstract to note the principle limitations and have also amended the main text following comments from the reviewers (see below) | Abstract  L 78-80 |
|  | Did your study have a prospective protocol or analysis plan? Please state this (either way) early in the Methods section. | There was a prospective protocol for collection of data, but not an analysis plan (which was not required by IRB or funding bodies). The protocol is included in supporting information as S2 Appendix | L 164, 191, S2 |
|  | If a prospective analysis plan (from your funding proposal, IRB or other ethics committee submission, study protocol, or other planning document written before analyzing the data) was used in designing the study, please include the relevant prospectively written document with your revised manuscript as a Supporting Information file to be published alongside your study, and cite it in the Methods section. A legend for this file should be included at the end of your manuscript. | As noted above no data analysis plan was made. | N/A |
|  | If no such document exists, please make sure that the Methods section transparently describes when analyses were planned, and when/why any data-driven changes to analyses took place. | We have reviewed the text for transparency and clarity; no data-driven changes to the analyses took place except describing the treatment (exclusion) of missing data. | L 230 |
|  | In either case, changes in the analysis-- including those made in response to peer review comments-- should be identified as such in the Methods section of the paper, with rationale. | We have reviewed the text for transparency | No changes |
|  | Please add the following statement, or similar, to the Methods: "This study is reported as per the Strengthening the Reporting of Observational Studies in Epidemiology (STROBE) guideline (S1 Checklist)." | We have added this text to the beginning of Methods section. | L 163-164 |
|  | Please include the completed STROBE checklist as Supporting Information. When completing the checklist, please use section and paragraph numbers, rather than page numbers. | Included as Supporting Information and moved to S1 Appendix as directed. | S1 Appendix file |
|  | In the methods, please describe how the adolescent questionnaire was developed and verified. Please provide | We have edited the text to say how the questionnaire was designed specifically for this study after reviewing literature and in consultation with local experts. | L199-202 |
|  | If you developed a questionnaire as part of this study and it is not under a copyright more restrictive than CC-BY, please include a copy, in both the original language and English, as Supporting Information, or include a citation if it has been published previously. | A copy has been included as Supporting Information S3 Appendix. | S3 Appendix file |
|  | In statistical methods, please refer to any post-hoc corrections to correct for multiple comparisons during your statistical analyses. If these were not performed please justify the reasons. Please refer to our statistical reporting guidelines for assistance | Corrections for multiple comparison were not made. The primary model was the Bayesian Network to identify the optimal model structure and it is not conventional to correct for multiple comparisons in such a model. | N/A |
|  | In statistical methods, please discuss how you accounted for clustering of repeated measurements in this cohort. | These analyses do not include repeated measures, since each adolescent was included only once and longitudinal data were summarized into a single observation (e.g., mean childhood HAZ or diarrhea incidence). | N/A |
|  | Your study is observational and therefore causality cannot be inferred. Please remove language that implies causality, such as “greater influence on”, “have greatly increased”, or “investments in education have unlocked...” Refer to associations instead. Please temper the last sentence of your conclusion to avoid overreaching what can be concluded from the data. For example, refer to phrases such as “education has the potential to unlock…” and so forth. | We have reviewed and amended text throughout the manuscript. | e.g.  Abstract, L558 |
|  | In the Methods and Results section |  |  |
|  | Please provide 95% CIs and p values for estimates in the main text and tables | We have added 95% CI and p-values where appropriate. | Results  e.g. L258-271, 313-333 |
|  | When a p value is given, please specify the statistical test used to determine it. | We have amended the text and Supporting Information Tables S1 and S3 to indicate the test used. | Tables S1, S3 and Results |
|  | Figures and tables: |  |  |
|  | Please indicate in the figure caption the meaning of the whiskers in Fig 4 | We have changed "lines" to "whiskers" for clarity. | Fig 4 (legend L 384) |
|  | Please define the following abbreviations in your tables. For example, IQR, SRCH, HAZ, BP, BMI | Thank you for this point, we have made edits accordingly to the Supporting Information Tables S1, S2, S4 and S5. | Tables S1, S2, S4 and S5 |
|  | The terms gender and sex are not interchangeable; please use the appropriate term. | Thank you for this important observation. We have edited the text following discussion with the editors. | L557 |
|  | Please use the "Vancouver" style for reference formatting and see our website for other reference guidelines | We have changed the references to match Vancouver style. | References |
|  | [https://journals.plos.org/plosmedicine/s/submission-guidelines#loc-references.](https://journals.plos.org/plosmedicine/s/submission-guidelines#loc-references) |  |  |
|  |  |  |  |
| **Reviewer 1** | **Page 8** The concept of a T score is not one widely used in health and I have seen it lead to confusion so perhaps add that it means standardised to mean 50 and standard deviation 10. | Thank you, we have added this to the text. | L216 |
|  | **Page 8** In fact Figure 3 seems to suggest that BMI was used as a continuous variable (which is the superior way to incorporate it) so perhaps this needs clarifying here. | We apologize for the confusion. We have clarified the text. | L223 |
|  | **Page 8** As written here anyone with systolic <120 would not be classified. Table 1 suggests otherwise. | Our apologies for the confusion. We have clarified the text. | L227 |
|  | **Page 9** If the score on the Raven’s is a count of positive responses as page 8 states then why re–scale it and then square it when a log linear model could be used? That would be the usual way of constructing a model for counts. | This is a good point. We were constrained by the statistical package used for the structure discovery (R package bnlearn) which does not currently accommodate log-linear models. We therefore transformed variables to approximate normality. | No changes |
|  | **Page 10** I am afraid I did not grasp the sentence starting ‘Of 126’. Figure 1 is clearer in this respect. | We have rewritten this sentence for clarity. | L264-265 |
|  | **Page 10** If the study participants have a mean age of 22.6 why use 19 in computing HAZ? I can guess but perhaps clarify. | We have edited the text to better explain. The WHO growth references only go up to aged 19 years. We updated Supporting Information Fig S1 and text. | Fig S1 (legend L727-730)  L284-286 |
|  | **Page 10** If you have adolescent HAZ why correlate childhood HAZ with adolescent height not HAZ? | The adolescent HAZ was an approximation as noted above because the maximum age for which the WHO have calculated a growth reference is 19 years. Rounding ages to this would unnecessarily introduce inaccuracies compared to using the observed height. | See cell above |
|  | **Page 11** Does this mean that adjusting for age and sex English speakers had had 2.4 years more education? As it is worded it looks as though it is saying people with equivalent actual years of education act as though they had 2.4 years more if they also spoke English. At least that is how I initially read it. | The former interpretation is correct. The 2.4 years of education was based on a linear model that adjusted for age and sex. Therefore, the interpretation is that adolescents who spoke English had, on average, 2.4 years more education than an equivalent adolescent matched for age and sex. We have amended the text for clarity. | L292-294 |
|  | **Page 12** As a very minor point I would write *t*–test in lower case to avoid any possible confusion with T–score. I assume Hotelling’s *T* ^2^ is not meant. | Thank you for this sensible suggestion. | Changes throughout |
|  | **Page 12** If the 49 people who do not speak Shina are unusual in some way I wonder whether any apparent relationship with that variable is driven by possible outlying values? | Thank you for this stimulating question. Perhaps surprisingly, the few non-Shina speakers were not substantively different (comparing the same set of variables that were used to look for bias due to loss to follow-up, q.v. S1 Table). There were slightly less males (35% [17/49] v 52% [729/1414]) and fewer had migrated to other cities (64% v 69%). This group also included all those who neither spoke Shina nor Burushaski (n=10) as a primary language. Given the lack of differences we have not made changes to the text. | No changes |
|  | **Page 13**, caption to Figure 2 The usual formula for BIC involved penal- ising minus twice the log likelihood by adding k ln n so I am not sure what log BIC would mean. | We apologize for the confusing sentence. The BIC from the model fitting was log-transformed to scale arcs for visualization purposes only, not for calculation of goodness of fit. The figure legend has been modified to reflect this. | Fig 2 (legend, L339) |
|  | **Figure 3** Is the *y*–axis the squared T–score or the raw score, or the T–score? | Thank you for pointing out this omission. We have modified Figure 3 accordingly. | Fig 3 modified |
|  | **Supplement S1** This reveals that what was used as the score was not the score on the original full scale. Without more information on the items which were dropped it is hard to assess how this affects the conclusions. Some of the appendix is going to be incomprehensible to people who are not familiar with administration of tests. I assume it is run in an adaptive way and if you fail a sequence of harder items you are auto– scored zero above that and vice versa for easy items but perhaps that can be clarified. | Thank you for raising this important point. We performed detailed psychometrics (as described in the Supporting Information S4 Appendix). That determined that a sub-set of items could be used, however this subscale was not so different from using the full set of items, which was also reliable. Our conclusion was that it was preferable to use the full Raven's Matrices, which is both externally validated and comparable to other studies. We have edited the Supporting Information Appendix S4 text to clarify this and noted the process of test administration. We have added a Supporting Information S8 Appendix Protocol on Raven’s Standard Progressive Matrices and Colored Progressive Matrices Administration | See Supporting Information S4 Appendix and S8 Appendix |
|  | **Supplement Table 4 and 5** I can see why some cells here are empty but do the others represent some form of variable subset selection? If so this needs reporting in the methods. Selecting a subset of variables in a way driven by the data leads to a model which is unlikely to replicate (Babyak, 2004). Screening variables to enter the model has been criticised too (Sun et al., 1996) | In an effort to group all of these models into a single (pair of) tables that thematically reflect the associations between diarrhea and pneumonia with key outcomes, we show the variables that are in all the models as rows. However, not all models shared the same suite of independent variables. The selection of what to include was based on the Bayesian Network and then we additionally include either diarrhea (Supporting Information Table S4) or pneumonia (Table S5). We have amended the table captions to reflect this. | Tables S4 and S5 and legends for each table. |
|  | Incorporating height and weight and BMI in the model seems rather strange given the artefactual relationship between them. The authors have excluded other variables from the network on theoretical grounds so I wonder why a theory–based choice was not made here. | BMI was an important outcome of interest (as an indicator linked to many chronic illnesses in later life) and so included. Equally, stunting in childhood was important and therefore included. However, height and weight were included in adolescence because of the connection between the two. It is true that the BMI necessarily incorporates both height and weight and that these arcs are essentially uninformative, however their inclusion does not detract from the overall model fit (or change other relationships). One nice feature of the Bayesian Network is that you can unpack relationships between variables and in this case we can begin to look at the distal drivers of BMI that might differentially relate to height and weight and offer greater insight than simply a relationship to BMI. | No changes |
|  | **Point of more substance**: The authors remark that there had been many positive social changes in the village in the intervening years and I note that the distribution of HAZ is much improved between time points. Excellent thing though this un- doubtedly is for the children is it another limitation to interpretation of the childhood predictors as they have been swamped by everyone being exposed to a more supportive environment? This would not alter the value of the study but it is perhaps worth mentioning. | Thank you for this interesting point. We have addressed this in reference to the comments on secular trends below. This is a complicated question because there are likely several phenomena occurring. First, is that there is a common phenomenon that prevalence of stunting decreases after 2yo. Leroy et al (10.3945/jn.114.191981) have partly attributed this to the heteroskedasticity height-for-age distribution, i.e. it is an artefact of using a relative rather than absolute measurement. Second, at least two studies (see Alam et al doi: 10.1371/journal.pone.0227839 and Crookston et al doi: 10.3945/jn.109.118927) have shown that children who are no longer stunted have cognitive scores that are no different to children who were not stunted. That seems likely in this population given the adolescent heights. The association between cognition and height is likely because both are constrained by similar factors in early life (e.g. nutrition – see the reviewer 2 comment below), but that these factors diverge as children get older (e.g. a nurturing and stimulating environment become more important to cognitive development). In this population there are substantial ‘background’ changes (e.g. incomes, jobs and education) but we still do not see differential effects between childhood factors (most notably HAZ and illness) and cognitive scores. This may be because the environment changed for everyone, but it appears that this ‘uncoupled’ any association that might have been expected given the childhood HAZs. We have added text to the discussion. | L435-446 |
|  |  |  | |
| **Reviewer 2** | Rasmussen and colleagues use longitudinal data show that investments in education of females is associated with adult human capital in a Pakistani village decades later. Research of merit and has broad implications. A few points. |  |  |
|  | Secular trends - How was this addressed in this work. Many populations improve in health/well being over time(in fact from S1, your cohort got healthier between childhood and adolescence). I am curious of how was these shifts were addressed in your analyses and how if might impact your overall findings. | This is a very interesting issue (q.v .‘social change’ above). In other work, e.g. Mansukoski et al [DOI 10.1017/S1368980019003239] identified secular trends in the health outcomes by looking at the same measurement in different birth cohorts of the same population in Guatemala. Such data are not available to demonstrate secular trends in the health outcomes for this study, but we can infer trends in per capita GDP by comparing regional reports and the clear difference between parents' and adolescent education experiences. We did not control for population trends, but were able to examine the effect of initial conditions and differential outcomes for individuals. The Bayesian Network also helps by unpacking the relationships between the many different factors to help interpret how (or not) some variables may confound associations. We have added text to the discussion. | Abstract & L441-4456 |
|  | The role of undernutrition in early life it seems to me is under-emphasized by authors. We know growth, brain development and other systems can all impact outcomes later in life -- all need proper nutrition. Similar work in Guatemalan birth cohort, and other LMICs showed that nutritional investments in females had impact on adult human capital and intergenerational benefits, with regards to offspring birth size. Underweight children have been found to be associated with failing a school grade in early life. It would be good if these points are discussed in this work to improve its contributory value. | This is a very important issue and we are grateful for the reviewer raising this. There are a very small number of longitudinal cohort studies and meta-analyses (e.g. Victora et al) have reported associations between growth and cognition that were not found here. One possibility is that this population has had greater social ("secular") changes, for example the targeted investment in school building and teacher training. These population-level effects may have ameliorated child-level limit factors (e.g. lack of stimulation) and broken the correlation between growth and development, outcomes that share similar drivers (most often relating to poverty) but are largely not causally linked. Our results are consistent with at least two other studies (e.g. Alam and Crookston - see new references 20, 21, 22) that show that recovery from stunting was associated with cognitive scores that were no different from children who were not stunted. We have added to the discussion. | Abstract & L435-446, 476-479 |
|  | Measurements - What was done to ensure reliability of clinical measurements(WT, HT, BP, WC etc) | Thank you for raising this important point. All measurements were taken by three trained Master’s level Field Coordinators. Where results were unexpected, measurements were repeated by the other Coordinator e.g. blood pressure. There was no formal testing of inter-observer agreement. | L220 |
|  | Raven score tests were not given to adolescents who had a phone interview. What proportion of the of your sample were these? How different were completers vs those who weren't interviewed? | Fewer than 1% of interviews were conducted by telephone. However, more broadly the issue of bias in those not sampled is an important point and we have added text to describe the differences in the 60 participants (most interviewed in person) who did not complete the Raven's Matrices (for any reason, but most often lack of time). | L266-271 |
|  |  |  |  |
| **Reviewer 3** | The study tries to examine the role of early childhood experiences on health, cognition, and education outcomes of adolescents/young adults in Gilgit Baltistan, Pakistan. Childhood data collected was compared with data collected in adulthood to examine the relationship. Strong introduction and methods sections. As the authors also highlight in line 64/65 that female empowerment and mental health variables were not available, yet the result section provides interesting insights. The manuscript has the potential to add to literature on examining the relationship of early and childhood health on adolescent/young adult health outcomes/status. |  |  |
|  | If available, reference number 5 needs to be updated to a recent study/report. | Thank you for prompting us to seek an update. The additional reference, World Bank. Pakistan Gilgit-Baltistan Economic Report: Broadening the Transformation [Internet]. Islamabad, Pakistan: World Bank; 2010 [cited 2021 May 18]. Available from: <https://portal.pnd.gog.pk/Content/Files/Reports/WB%20Economic%20Report-GB%20pdf_200512092.pdf> is a valuable point in addressing the context of secular trends. | L149 |
|  | Reference numbers for the ethics approval need to be reported in ethics approval section. | In the 1980s and early 1990s, the Aga Khan University did not have protocol numbers but dates of study approval are noted. The relatively new Karakoram International University convened a special, ad-hoc Ethical Review Committee for this protocol and it was not numbered. | L168-175 |
|  | Although authors discuss bias in lines 423-424, some discussion on recall bias needs to be reported as a limitation for SRPH. | We apologize for this oversight and have added it to the abstract and discussion sections. | Abstract L79-80 & L541-542 |
|  |  |  |  |
| **Reviewer 4** | This well-structured manuscript describes an important study which investigated the non-verbal cognitive outcomes of a group of adolescents and young adults followed up following an early life cohort study of under-5 mortality and morbidity in Pakistan. This study leverages the value created by a large initial cohort study from the 1990's and spans of period of rapid socio-economic mobility due to increased accessibility of one of the poorest regions in Pakistan. While the high level results reported here of higher SES (in particular, maternal education), predicting better cognitive outcomes are not highly novel, they are nevertheless important given the region on which the study reports, the authors do include discussion on the context specific elements at a more granular level which are highly relevant and important in thinking about potential of such findings to inform policy...(comments below)... No major revisions needed from my perspective, though suggest a formal statistical review and publication of analysis plan if possible |  |  |
|  | The inclusion of measures of physical health (growth metrics, BMI, hypertension) as signals of potential risk for future NCD's is an additional strength. The methods and analysis approach is well described including specific reasons for children lost to follow up. | Thank you. We would like to be able to continue to follow up this cohort in the future for development of NCDs, research personnel and funding permitting. |  |
|  | As noted in the abstract, measures on maternal mental health does seem like an important limitation and the potential role that this may have played deserves some discussion in the limitations section. | Thank you for noting this important limitation. We have amended the text. | L535 |
|  | Further discussion on the critical role this period of life represents in terms of being the crucible for intergenerational risk and resilience would add value. | We appreciate this point, both because of the importance of early childhood and of adolescence. We have added text to the discussion. | L468-469  & L425 |
